# Bayesian weighting of climate models based on climate sensitivity

**DOI:** 10.1038/s43247-023-01009-8

**Published:** 2023-10-20

**Authors:** Elias C. Massoud, Hugo K. Lee, Adam Terando, Michael Wehner

**Affiliations:** 1 Computational Sciences and Engineering Division, Oak Ridge National Laboratory, Oak Ridge, TN USA; 2 Jet Propulsion Laboratory, California Institute of Technology, Pasadena, CA USA; 3 U.S. Geological Survey, Southeast Climate Adaptation Science Center, Raleigh, NC USA; 4 Department of Applied Ecology, North Carolina State University, Raleigh, NC USA; 5 Applied Mathematics and Computational Research Division, Lawrence Berkeley National Laboratory, Berkeley, CA USA

**Keywords:** Projection and prediction, Environmental impact

## Abstract

Using climate model ensembles containing members that exhibit very high climate sensitivities to increasing CO_2_ concentrations can result in biased projections. Various methods have been proposed to ameliorate this ‘hot model’ problem, such as model emulators or model culling. Here, we utilize Bayesian Model Averaging as a framework to address this problem without resorting to outright rejection of models from the ensemble. Taking advantage of multiple lines of evidence used to construct the best estimate of the earth’s climate sensitivity, the Bayesian Model Averaging framework produces an unbiased posterior probability distribution of model weights. The updated multi-model ensemble projects end-of-century global mean surface temperature increases of 2 ^o^C for a low emissions scenario (SSP1-2.6) and 5 ^o^C for a high emissions scenario (SSP5-8.5). These estimates are lower than those produced using a simple multi-model mean for the CMIP6 ensemble. The results are also similar to results from a model culling approach, but retain some weight on low-probability models, allowing for consideration of the possibility that the true value could lie at the extremes of the assessed distribution. Our results showcase Bayesian Model Averaging as a path forward to project future climate change that is commensurate with the available scientific evidence.

## Abstract (web summary)

The probability distribution of future global mean surface temperature and its uncertainty can be estimated using a Bayesian Model Averaging framework, thereby alleviating the so-called hot model problem without rejecting any models from the ensemble.

## Introduction

Numerous climate modeling groups submitted coordinated experiment output to the latest round of the Coupled Model Intercomparison Project, phase 6 (CMIP6)^1^. These climate model outputs are used to inform national (e.g., Fifth U.S. National Climate Assessment, NCA5) and international assessments (e.g., Intergovernmental Panel on Climate Change Sixth Assessment Report, or IPCC AR6) regarding climate change and its societal impact. While the information provided by CMIP6 models is critical for understanding the consequences of anthropogenic greenhouse gas emissions and projecting future climates, many of the models are considered ‘too hot’^2^), meaning they simulate a warming response to the change in radiative forcing that is too strong given other lines of evidence and our physical understanding of the climate system^3–8^.

In response to this, various approaches have been proposed to construct a climate model ensemble of transient projections that are more consistent with assessed estimates of the Earth’s climate sensitivity. These range from very rigorous and extensive approaches to simple model culling exercises. For example, the report submitted by the Working Group I (WG1) to the IPCC AR6, combined multiple lines of evidence from observations, theory, and the use of physically based energy-balance model climate emulators to constrain 21st century global surface air temperature projections^9^. The lines of evidence used in the IPCC AR6 include feedback process understanding, climate change and variability seen within the instrumental record, paleoclimate evidence, and so-called ‘emergent constraints’, and importantly do not include estimates of climate sensitivity from the climate models. On the other end of the spectrum, Hausfather and colleagues^10^ have suggested a method to reject CMIP6 model projections of transient warming if they are outside the bounds of the IPCC AR6 assessed likely range of Equilibrium Climate Sensitivity (ECS) or the Transient Climate Response (TCR). They showed that the ensemble mean projected global mean surface temperatures of such a subset of CMIP6 models are closer to what is assessed in the IPCC AR6 compared to the entire CMIP6 ensemble. Other studies are beginning to emerge that apply similar methods of model rejection, or model culling, to reduce the importance of the ‘hot models’ in their climate change projections^11^.

The attractiveness of the model culling approach lies in its simplicity, given that the WG1 method of using model emulators is unlikely to be easily replicated across the numerous contexts in which CMIP6 model outputs are now used. Furthermore, it acknowledges that the use of the recently popularized alternative approach known as ‘Global Warming Levels’^12^, is not likely to meet the needs of many decision makers who are increasingly reliant on transient climate simulations to assess time-dependent risks (i.e., it is not enough to know *what* the consequences of 3 °C global warming are, but also when they are likely to occur, conditional on future emissions pathways). A notable drawback of the model culling approach, however, is that rejecting models is akin to applying a binary weighting scheme to the CMIP6 ensemble, with zero weight applied to the culled models, and model democracy^13^ for the remaining ensemble members. Thus, while simple and easy to implement, this somewhat heavy-handed approach results in the potentially unsatisfactory outcome of eliminating consideration of the information provided by a significant portion of the ensemble^14^.

Here we provide an alternative approach to construct a weighted CMIP6 ensemble projection that is consistent with the IPCC AR6-assessed range of ECS and TCR, as well as the culled ensemble work shown in ref. ^10^, without having to force the exclusion of high-sensitivity models. We use Bayesian Model Averaging (BMA)^15–20^ as a framework to constrain the CMIP6 ensemble projection based on the IPCC-assessed range of ECS or TCR values, allowing information from all considered models to be included in the final projection. In short, BMA is a method that tries thousands of combinations by sampling different model weights and compares the created model averages with a desired target field, in this case a realistic ECS or TCR value. After sampling thousands of combinations, the posterior combinations (or the most optimal sets of model weights) are extracted and used for post-processing. This ultimately avoids having to reject any models that are considered ‘too hot’, since all models may appear in any given set of weights.

Applying BMA in this context is a novel strategy that grants all the models in the ensemble to ‘have a voice’ and provide information to the estimated model average of ECS or TCR. Therefore, this strategy is an advancement from simply eliminating ‘hot models’ from the ensemble, as other studies have done and are currently doing^10,11^. Furthermore, the novelty of this study is to apply Bayesian model weighting to solve the ‘hot model’ problem, or more generally to calibrate a set of models to a desired distribution of sensitivity, whereas previous works that have applied model weighting have calibrated directly to observations such as historical temperature or precipitation^18,20,21^. The value here is that ECS and TCR are much more relevant for future climate change than are past historical temperature observations, so constraining models based on sensitivity will be more meaningful in capturing simulated future climates (provided that the ECS and TCR distributions are strongly informed by relevant observations and evidence). Given the recent focus on the high sensitivity of some CMIP6 models, a plausible outcome is that whichever approach is adopted to address this issue is then applied to projections of other fields (e.g., precipitation). Thus, our procedure is partially motivated by a desire to preserve as much information as possible from the ensemble that is consistent with our physical understanding of the earth’s climate system.

## Results and discussion

### The community’s evolution with model weighting strategies

In the last 20 years, there has been a transition from using simple multi-model means to using weighted ones^20,22–29^. The central idea is that with enough information to determine a weight for each model, the projections based on model weights derived from the model evaluation against observations have been shown to have greater accuracy than an arithmetic multi-model mean, and this has been determined in many studies^13,22,25,30^. For many cases, Bayesian approaches were used to determine the model weights^4,17–20,22,31–36^.

More recently, weighting based on model independence has been an additional criterion to consider alongside model skill. This consideration of model independence has emerged due to models having common bases of model structure, parameterizations, and associated programming code, all of which can result in a lack of independence between climate models^13,17,18,21,37,38^. Earlier works utilized model skill and independence in empirical formulas that determined the model weights based on vectorized information of the skill and independence that were used as inputs to the weighting equations^21,37–39^. Instead of using empirical formulas, ref. ^18^ showed that independence information can be estimated in the post-processing of the model weighting exercise, and they determined model independence information using the posterior distribution of the BMA weights estimated in their studies. This was also done in ref. ^19,20^, where many variations of model weighting strategies that utilized model skill and independence information were implemented and compared.

### Individual model sensitivity

**Fig. 1:**
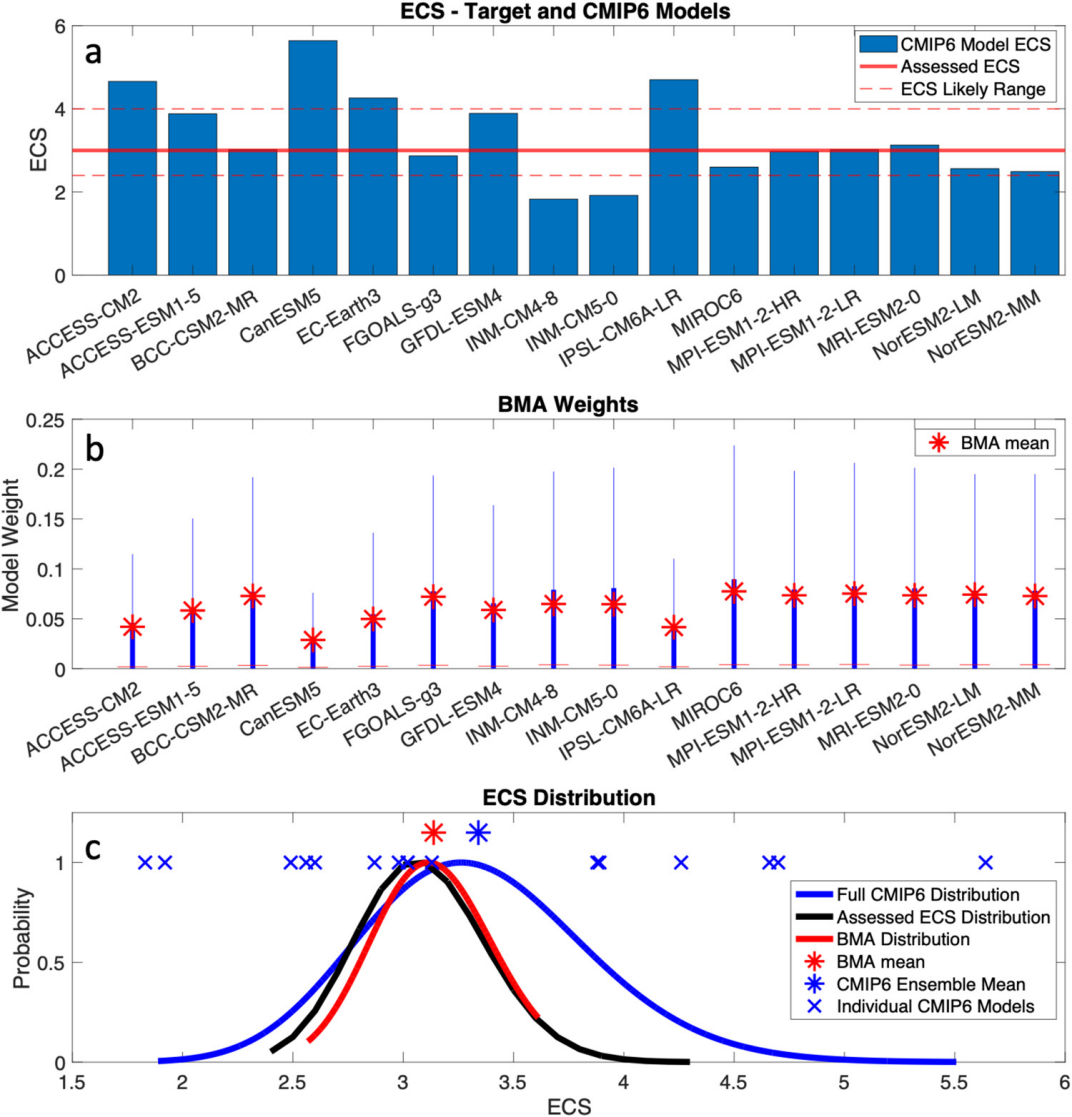
Model weighting using ECS as the main fitting target. **a** Equilibrium Climate Sensitivity (ECS) for 16 Earth System Models (ESMs) from the CMIP6 archive. The red line here depicts the IPCC assessed central value estimate of ECS, which is 3 °C (dashed red lines show the upper and lower bounds of the assessed ECS distribution). **b** BMA posterior distributions (blue box-and-whisker plots) of the model weights after using the assessed ECS distribution as a fitting metric, with the mean BMA weights shown with the red stars. **c** The ECS value from each CMIP6 model (blue x) with the distribution from the raw CMIP6 ensemble estimated from Monte Carlo sampling of the model weight space (blue curve), the target assessed ECS distribution (black curve), and the final BMA estimated posterior distribution of ECS (red curve).

ECS is an important quantity used to estimate how the climate responds to radiative forcing and is an estimate of the eventual steady-state global warming given a doubling of atmospheric CO_2_ concentrations. Based on multiple lines of evidence, the IPCC AR6 assessed best estimate of ECS is 3 °C with a likely range of 2.5–4 °C (high confidence)^40^. This estimate from AR6 is used as the target distribution for ECS in our study and is shown as the black curve in Fig. 1c. Individual CMIP6 models are expected to simulate a similar climate sensitivity, yet some models are below, and other models are well above this range^41^. Table 1 lists a set of 16 models and their ECS values (also shown in Fig. 1a) from the CMIP6 archive that are common to two forthcoming statistically downscaled datasets to be used in scientific impact and assessment activities across North America. The approach taken in ref. ^10^, when transient simulation output is required, is to eliminate any models that are outside the likely range of ECS (2.5–4 °C). Here, we propose an alternative approach, which is to apply BMA and find an appropriate linear combination of models that produces a good fit to the likely ECS distribution.

**Table 1:** Individual model weights and dependence scores based on ECS and TCR.

Model Name	ECS	BMA Weights based on ECS	Dependence based on ECS	TCR	BMA Weights based on TCR	Dependence based on TCR	ECS Screen	TCR Screen
ACCESS-CM2	4.72	0.0412	−0.204	1.96	0.0708	0.0618	High	
ACCESS-ESM1-5	3.87	0.0581	−0.0238	1.97	0.0715	0.0715		
BCC-CSM2-MR	3.04	0.0723	0.1029	1.55	0.0639	0.0288		
CanESM5	5.62	0.029	−0.3802	2.71	0.0494	−0.2283	High	High
EC-Earth3	4.3	0.0498	−0.1122	2.3	0.0626	−0.0529	High	High
FGOALS-g3	2.88	0.0716	0.0712	1.5	0.0608	−0.0075		
GFDL-ESM4	3.9	0.0589	0.0042	1.63	0.0688	0.0569		
INM-CM4-8	1.83	0.0646	−0.1322	1.3	0.0501	−0.1065	Low	Low
INM-CM5-0	1.92	0.0649	−0.0809	1.41	0.0552	−0.0395	Low	
IPSL-CM6A-LR	4.56	0.0449	−0.1757	2.35	0.0614	−0.0661	High	High
MIROC6	2.61	0.0767	0.0947	1.55	0.0664	0.0407		
MPI-ESM1-2-HR	2.98	0.0731	0.088	1.64	0.0686	0.0595		
MPI-ESM1-2-LR	3	0.0755	0.1333	1.82	0.0737	0.1195		
MRI-ESM2-0	3.15	0.073	0.1289	1.67	0.0712	0.0919		
NorESM2-LM	2.54	0.0736	0.087	1.49	0.0596	0.0049		
NorESM2-MM	2.5	0.0727	0.0551	1.22	0.0461	−0.1591		Low
Ensemble Mean	3.33875			1.7544				
BMA mean	3.1431			1.7534				

This table lists models from the CMIP6 ensemble that are considered in this study. The ECS and TCR values for each individual model are listed, along with the BMA mean weights (based on ECS or TCR), and the dependence score estimated from the BMA posterior distributions (also based on ECS or TCR). The two columns on the right depict whether a model is higher or lower than the assessed ECS or TCR range (models that have a high or low ECS or TCR were rejected in Hausfather et al.’s method). The 16 models included here are those included in the NCA 5th report. All models included here are also included for all future scenarios.

By searching for various combinations of these CMIP6 models that best fit the ECS distribution, rather than culling the models that are ‘too hot’, we recast the information signal from each model to varying degrees. These results are displayed in Fig. 1. Each individual model’s ECS is shown graphically in Fig. 1a with a red line indicating the mean target ECS value of 3 °C. Figure 1b shows the BMA posterior distribution of weights that are estimated for each model, with the mean of these distributions also listed in Table 1. In essence, out of 15,000 samples of model combinations, the distributions shown in Fig. 1b utilize 2/3 of the model weights from the posterior samples, which allows the best fit to the target ECS value and the expected ECS distribution, shown in Fig. 1c. This enables the creation of a weighted ensemble that is consistent with the assessed probabilistic uncertainty around the true (and likely unknowable) ECS value (distributions in Fig. 1c).

**Fig. 2:**
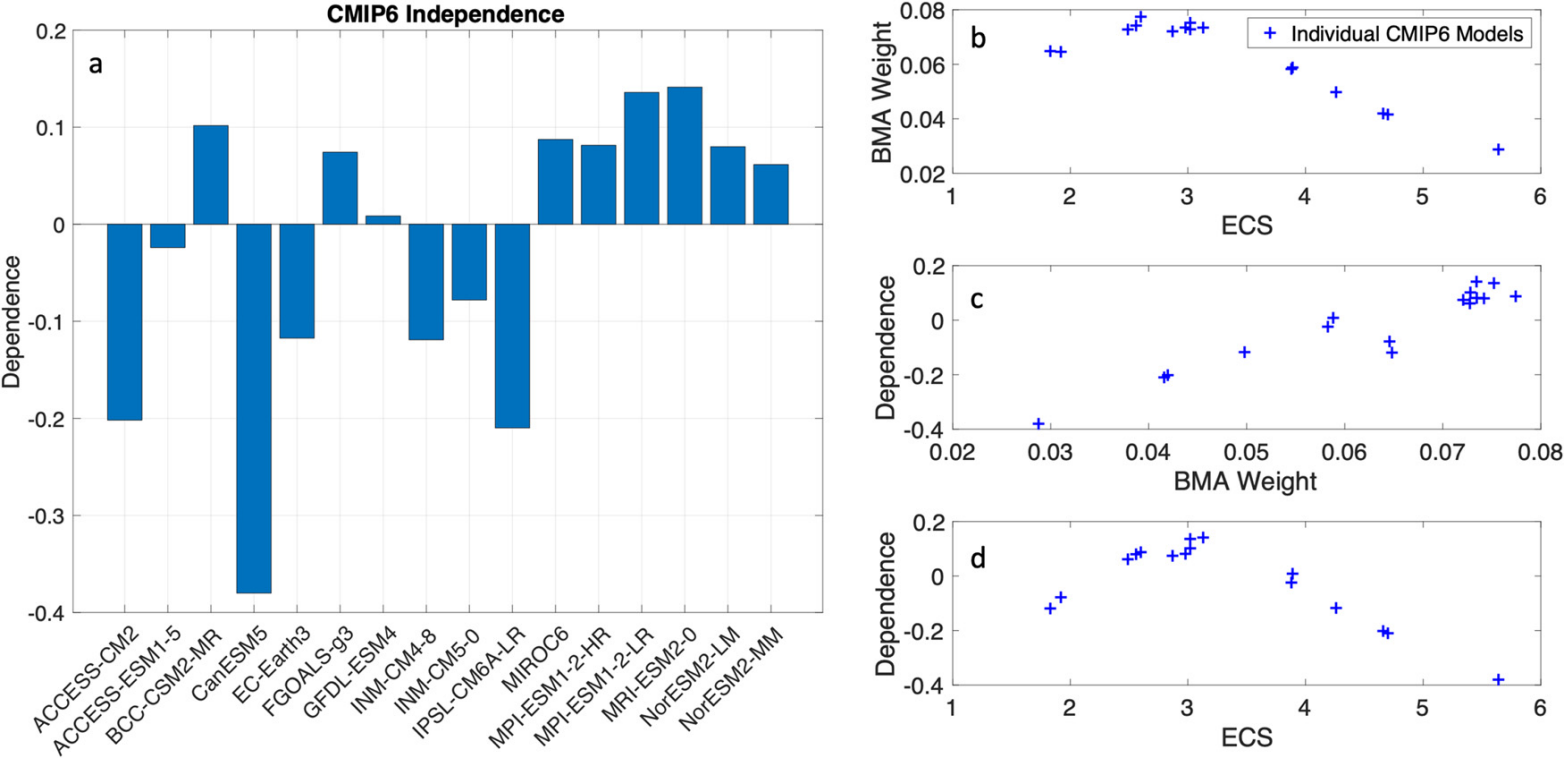
Relations between model weight, independence, and ECS scores. **a** This plot shows the bar graph of the model dependence scores estimated from the BMA posterior distributions when using the ECS as a fitting metric. A higher (more positive) value indicates a model with higher dependence on other models (i.e., a less independent model), while a lower (more negative) value indicates a model with less dependence (i.e., a more independent model). These panels show scatter plots of each individual CMIP6 model and the relationship between (**b**) the BMA weight and the corresponding ECS value, (**c**) the dependence score and corresponding BMA weight, and (**d**) the dependence score and corresponding ECS value. This figure highlights how models that are ‘too hot’ have lower BMA weights and dependence scores, and this decrease in weight drops almost linearly with increasing ECS value.

What is more, model independence is a desired trait when applying any kind of model averaging^18–21,37,39^. The BMA posterior samples can be used to estimate a level of independence that each model is offering to the model average. The independence scores for each model are listed in Table 1. Unlike other methods that apply independence as a predetermined metric^37,39^ independence here can be calculated after post-processing the posterior BMA weights^19–21^ (Fig. 2a). Generally, models with a high ECS tend to receive lower weights, and models with lower weights also have lower dependence scores, and therefore, models with high ECS also tend to have lower dependence scores (Fig. 2b–d). Furthermore, the BMA model weights tend to drop linearly as the model ECS value moves away from the peak estimated ECS of 3 °C (Fig. 2b). This result shows that models outside the likely range of 2.5–4 °C tend to have lower model weights.

**Fig. 3:**
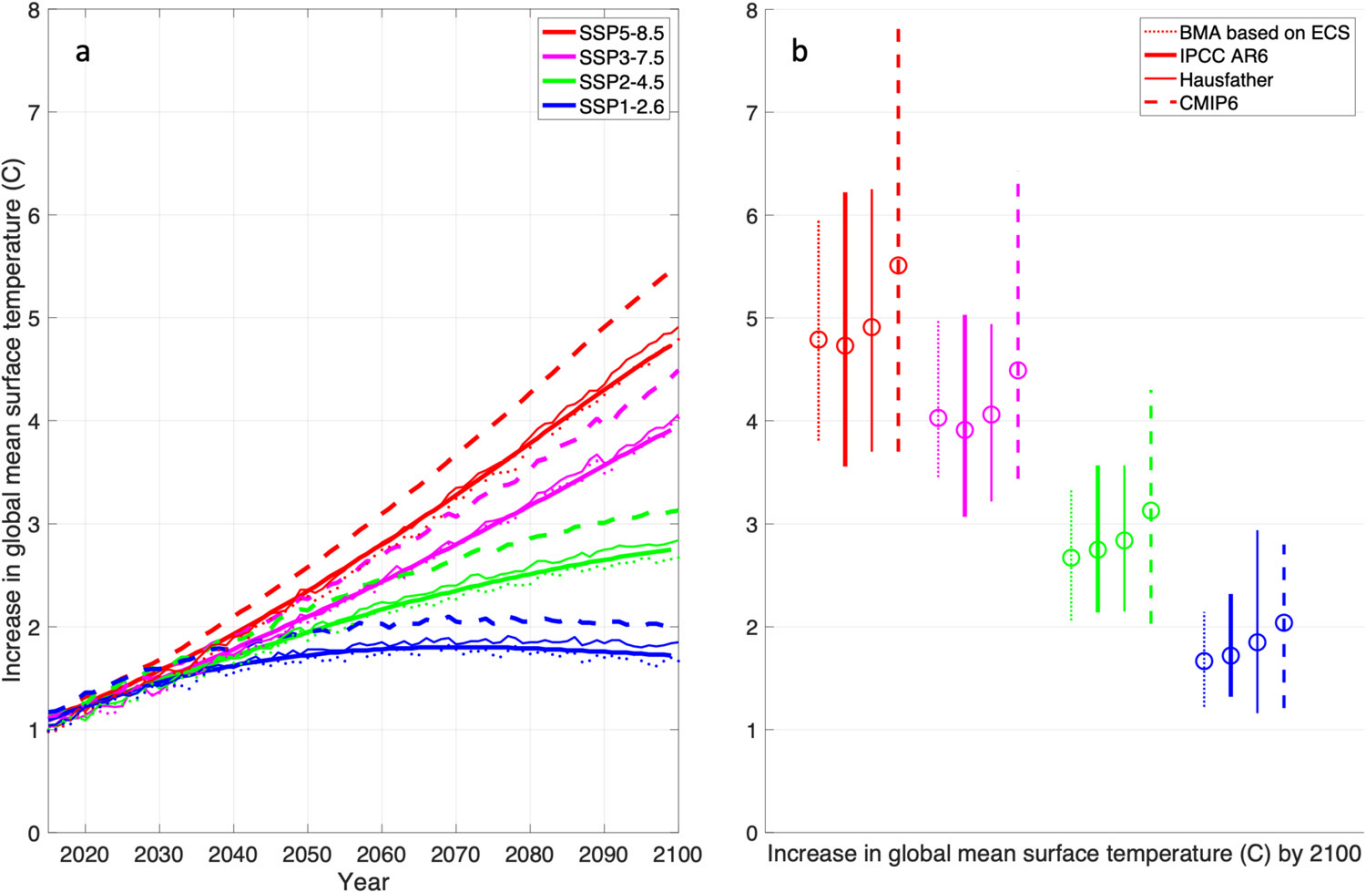
Future projections of global mean surface temperature based on ECS. **a** Increase in global mean surface temperature (°C) for the different SSP scenarios considered and the different model averaging methods used. Dashed lines are the raw CMIP6 mean, light solid lines are from Hausfather et al., dark solid lines are the AR6 assessed warming levels, and dotted lines are the results produced in this paper from the BMA method when using ECS as a fitting metric. **b** Increase in global mean surface temperature by the year 2100 and the uncertainty ranges of this estimate for each SSP scenario and each model averaging method considered here. Results shown here have no temporal filtering. The BMA uncertainty bar plotted here is the top 95% of the full posterior distribution of model weights.

When the set of posterior weights are applied, the result is a weighted ensemble mean for each Shared Socioeconomic Pathway (SSP1-2.6, SSP2-4.5, SSP3-7.0, and SSP5-8.5) that lies very close to both the IPCC assessed best estimate and the ref. ^10^ culled multi-model mean. The results showing the future global mean surface temperature projections are shown in Fig. 3a. Notably, the 95% uncertainty range of the end-of-century temperature increase signal, shown in Fig. 3b, is also consistent with the IPCC-assessed range for the four considered SSPs, whereas the model-culling method still results in a wider ensemble range for the SSP1-2.6 scenario.

### Benefits of model weighting

The different emission scenarios (SSP1-2.6, SSP2-4.5, SSP3-7.0, and SSP5-8.5) are clear in what they project for the future. In short, expected temperature changes increase as the projected emissions increase. However, the benefits of the different methods used to combine CMIP6 models is not so clear. For example, prior to the IPCC AR6, most assessments simply used the arithmetic mean of multiple models, which can lead to higher projections of warming than what is realistically possible, since some of the models in the CMIP6 ensemble are deemed ‘too hot’. Therefore, the IPCC AR6 ‘assessed warming’ trends are obtained by applying statistics to determine the most reasonable projections that are consistent with multiple lines of evidence for metrics such as ECS and TCR. Furthermore, works such as ref. ^10^ simply reject models that fall outside the likely ECS or TCR range, and create new multi-model mean trends from the subset of models that have not been eliminated.

The strategies taken by the IPCC AR6 and in works like ref. ^10^ produce new future climate estimates that are less exaggerated than those produced using the raw CMIP6 multi-model mean, as shown in Fig. 3. While the results from Hausfather et al. move the curve closer to the IPCC AR6 trends, they do so at the expense of rejecting some models (rejected models from their work are pointed out in our Table 1, right columns). Whereas the BMA approach maintains all the models in the ensemble and achieves very similar results (see Fig. 3), both for the mean signal as well as for the structural uncertainty of the estimate. This is achieved by applying lower weights for models with ECS that are considered either too low or too high (as shown in Fig. 2b) and applying heavier weights for models that are closer to the expected ECS value.

The original spread of the CMIP6 signal for end-of-century change in global mean surface temperature is much broader (almost twice the spread) compared to the estimate when ‘hot models’ are downweighed or eliminated from the ensemble (i.e., Fig. 3b). The distribution from the original CMIP6 ensemble is usually skewed in a way that makes the mean signal much higher than for the methods that ameliorate the ‘hot model’ problem (e.g., ~5.5 °C for SSP5-8.5 compared to below 5 °C, Fig. 3b). Therefore, when the ‘hot model’ problem is addressed, either by down-weighting, or culling, future global mean temperatures are lower, and increases in temperature are less exaggerated. Yet, the distribution of this estimate is not always ameliorated in the same way.

Figure 3b shows the spread of each method’s future signals, with both BMA and the IPCC AR6 assessed warming exhibiting close alignment across all SSP scenarios, whereas the Hausfather method of culling the hot models has a similar spread for most scenarios, except for SSP1-2.6 which has an even higher spread than the original CMIP6 ensemble. This increase in spread is an odd finding in the ref. ^10^ effort, and we hypothesize that it could be because by culling, or by eliminating, models that have a high ECS value, and therefore by eliminating models that are ‘too hot’, there is also an elimination of information of how these models simulate climate in a scenario that has little increased warming, i.e., the SSP1-2.6 scenario. This reinforces the idea that simply rejecting models in an ensemble may not be the optimal or ideal way to conduct a model averaging problem, since model culling may work for ‘hotter scenarios’ but might break down for ‘cooler’ ones. We argue that the special benefit of BMA is that it provides posterior information that provides probability densities on the weights of each individual Earth System Model (ESM) in an optimal manner, which allows all the models in the ensemble to provide accurate information to the model average.

### ECS and TCR

Our approach makes use of the ECS metric as a main target for fitting the BMA model average. However, other metrics can be useful in this regard as well, such as the TCR, which is the mean global warming predicted to occur around the time of doubling CO_2_ in ESM runs for which atmospheric CO_2_ concentration is prescribed to increase at 1% per year. Based on multiple lines of evidence^41^, TCR has an assessed likely range of 1.4–2.2 °C (c.f. the IPCC AR6 WG1 technical summary). Therefore, we apply the BMA on the assessed TCR distribution to produce a set of weights that optimize the model based TCR estimate.

We produced a second set of results to mirror the outcome from applying BMA on the ECS metric (the results of which are shown in the main text, Figs. 1–3, Table 1), but we did so for the TCR metric (the results of which are shown in the supplement, Figs. S1–S3, Table 1). Like Fig. 1 that is based on the ECS metric, supplemental Fig. S1 shows results based on the TCR metric, including individual CMIP6 model TCR scores, the assessed TCR distribution, and the estimated BMA model weights and corresponding TCR distributions. Out of 15,000 samples of model combinations for TCR, the distributions shown in Fig. S1B utilize 3/4 of the model weights from the posterior samples, which allows the best fit to the target TCR value and the expected TCR distribution, shown in Fig. S1C. Supplemental Fig. S2 shows the dependence scores based on applying BMA on the TCR metric, as well as how these scores relate with the BMA model weights and TCR scores. The final figure in the supplement, Fig. S3, shows the projected increase in global mean surface temperature (°C) for the different SSP scenarios considered and the different model averaging methods used, and panel B of this figure includes both the ECS (black dotted lines in Fig. S3B) and TCR-based projections (colored dotted lines in Fig. S3B) for comparison and shows that the results do not differ much between the two.

Like the results of applying BMA on ECS, doing so on the TCR metric produces a weighted mean projection that is more in line with all lines of evidence compared to that produced using the raw CMIP6 multi-model mean. The results show that some models are indeed ‘too hot’, but there are also models in the ensemble that are ‘not hot enough’, meaning they have an ECS or TCR value that is lower than the assessed range. So, the BMA method downweighs models that are either ‘too hot’ or ‘not hot enough’. This is displayed in the scatter plots in Fig. 2b–d and Fig. S2B–D, which show the highest weights are applied for those models with ECS and TCR values near the peak (near 3 °C for ECS and 1.8 °C for TCR), and the weights do drop linearly for ECS and TCR values that are higher or lower than the peak value. However, even though the weights drop for some models, there are no models that have weights that are too low or too high. In other words, all models generally have weights in the range of ~0.03–0.08. For comparison with the equal weights approach (i.e., a simple model mean), all models would have a weight of 1/16 or 0.0625 if equal weights were applied. Again, this is the benefit of using BMA over the other mentioned methods since all models can still provide significant information to the model average when using the BMA method. Moving forward, in climate assessment reports as well as for other scientific analysis, we recommend the use of model weighting (e.g., BMA-based methods) on metrics such as ECS or TCR. This will facilitate a more rigorous calibration of information that is used from models that are ‘too hot’ without having to outright reject them from the ensemble.

While our approach is based on global mean temperature changes, we suggest that these weights also could be used to estimate projected changes and uncertainty in other fields, such as precipitation. In other words, the BMA weights based on ECS or TCR can be used to make projections for any climate field, where the benefit would be that the response of these fields to temperature would be accounted for, but the drawback is that there would be no information about the quality of these fields and therefore an evaluation would need to take place. We note that IPCC AR6 also considered projections at “global warming levels” at 1.5, 2.0, 3.0 and 4.0 °C above preindustrial levels. While the high sensitivity models arrive at these levels too quickly, all models were included in estimates of projected changes in the IPCC AR6. We suggest that model weighting can also be used to make weighted estimates of when these global warming levels are reached.

Overall, allowing for the use of the full suite of state-of-the-science ESMs to help inform societal responses to anthropogenic climate change, rather than subjectively picking some out of the ensemble and rejecting others, should be the preferred path forward when estimating future climate change projections and their impacts.

## Conclusions

In conclusion, as an alternative to simple model culling, we recommend the use of any model averaging approach that allows the user to justify non-zero weights on all members of the model ensemble. In this study, we have focused on ECS and TCR to address the hot model problem. Previous studies targeted towards specialized impact studies focused on other phenomena such as drought^42^ or used an expert but arbitrary selection of observed mean quantities as in ref. ^39^ targeted towards a skillful general-purpose ensemble (we refer to this as the Sanderson approach). However, the unreasonably large range of model climate sensitivities in the CMIP6 ensemble requires attention to model trends. Here we have used a BMA approach, but previous statistically based model weighting studies have used a simple kernel-density estimation (KDE) approach^42^ or the Sanderson approach^39^. With KDE, each ESM is weighted by the ratio of the target density to the local sample density of models in ECS or TCR space. The Sanderson approach implements the predetermined skill and independence scores of each model when estimating the model weights.

We particularly recommend BMA to apply model averaging when feasible, since the total-order distribution of the model weights (and therefore the total-order distribution of the projected climate change signal) is estimated with BMA, whereas with the KDE or Sanderson approaches, only the first-order distribution of the model weights is estimated. In other words, the BMA method allows for the estimation of the full distribution of model weights given the evidence (i.e., the IPCC assessed distributions) and therefore the full uncertainty distribution, whereas the KDE or Sanderson approaches nudge the model weights in the direction of the optimal set of weights without the estimation of the distribution. Furthermore, regarding model independence, the BMA method allows for the estimation of model independence during post-processing and provides information on dependent model weights that are used for future projections. This is important because we want to know the dependence of each models’ contribution to the estimated model average (and therefore the dependence of each models’ contribution to the projected climate change signal with BMA). In comparison, the Sanderson approach uses predetermined information to estimate model independence and is based on the model output space, not the model weights space. This is different than the BMA estimation of model dependence because the Sanderson approach uses information on which models have similar model genealogy, shared code and parameterizations, or similar model outputs, while BMA provides information on which models have posterior weights that are correlated together (and therefore which models are dependently contributing to the projected climate change signal). What is more, the KDE approach does not estimate model independence. For these reasons, we highly recommend the use of the BMA method for model averaging studies in climate sciences.

## Methods—Bayesian Model Averaging (BMA)

BMA is different from other model averaging methods because it explicitly estimates each model’s weight and associated uncertainty by maximizing a likelihood function that represents the fit to the target distribution. In other words, BMA provides model weights that produce linear model combinations of multiple models, and these combinations have a higher likelihood of matching the target data compared to other model combinations. In this study, using the optimized weights, BMA constructs the mean and uncertainty distribution of the ECS (or TCR) metric.

The estimated model weights using BMA are defined as ${{{{{{\bf{w}}}}}}}_{i,{{{{{\rm{BMA}}}}}}}=\left[w\left({m}_{1}\right),w\left({m}_{2}\right),\ldots ,w\left({m}_{k}\right)\right]$, for K models. In our case, *K* = 16. The range of $w\left({m}_{i}\right)$ is between 0 and 1, with a weight of 0 for models that do not contribute any information and a weight of 1 for models that fully contribute to the estimation. The sum of any given combination of model weights is equal to 1. The final estimate of the BMA model weights, or ${{{{{{\bf{w}}}}}}}_{i,{{{{{\rm{BMA}}}}}}}$, are utilized to constrain the ECS (or TCR) distribution generated by the model average as well as the spread of uncertainty in the projected climate change signal.

The likelihood for each of the sampled model weights depends on how the generated ECS (or TCR) value from the combined model average compares with the target ECS (or TCR) distribution. The prior distribution of model weights is initialized as a Jeffreys prior^43^ which transforms the normalized prior distribution of model weights from a non-informative uniform distribution covering a smaller range of the model weight space to an informative distribution covering a larger range (this ensures that model weights are sampled from 0 to 1). The target ECS distribution is assumed to follow a gamma distribution with parameters *a* = 67.696 and *b* = 0.0476, which results in a target ECS distribution with a range of 2.5–4 °C and a peak near 3 °C (similar to the likely range of ECS reported by the IPCC AR6). This target ECS distribution is shown with a red curve in Fig. 1c, labeled as ‘Target Distribution’. Then, the expected probability for the ECS values generated by the different BMA sampled weights, i.e., ${{{{{{\rm{ECS}}}}}}}_{{{{{{\rm{CMIP}}}}}}}({{{{{{\bf{w}}}}}}}_{i,{{{{{\rm{BMA}}}}}}})$, is estimated using the gamma distribution described above. This probability distribution is used to inform the likelihood function in the BMA framework, i.e., $L({{{{{{\bf{w}}}}}}}_{i,{{{{{\rm{BMA}}}}}}})$, and this likelihood function is maximized in search for the optimal set of model weights (or optimal set of model combinations). Therefore, the likelihood function becomes proportional to the difference between the BMA generated ECS value and the target ECS distribution, i.e., $L({{{{{{\bf{w}}}}}}}_{i,{{{{{\rm{BMA}}}}}}})$ ∝ [${{{{{{\rm{ECS}}}}}}}_{{{{{{\rm{Target}}}}}}}$ – ${{{{{{\rm{ECS}}}}}}}_{{{{{{\rm{CMIP}}}}}}}({{{{{{\bf{w}}}}}}}_{i,{{{{{\rm{BMA}}}}}}})$].

The same likelihood formulation can be applied for the TCR metric, which has a target TCR distribution that follows a gamma distribution with parameters *a* = 119.734 and *b* = 0.0147. This results in a target TCR distribution with a range of 1.4–2.2 °C and a peak near 1.8 °C. The target TCR distribution is shown with a red curve in Fig. S1C of the supplementary section.

For each test (ECS and TCR), we apply heavy sampling (*n* = 15,000 samples) on the possible model weight combinations in search of model weights that maximize the likelihood functions described above. This allows for the estimation of the optimized BMA model weights, or ${{{{{{\bf{w}}}}}}}_{i,{{{{{\rm{BMA}}}}}}}$, shown in Fig. 1b for ECS (and in Fig. S1B in the supplementary section for TCR).

Since the BMA method estimates a distribution of model weights, N (N»1) model combinations become possible, which provides a solution to the model dependence issue. In other words, consider that in the BMA framework there is a hypothetical Model-1 and a Model-2 that are similar and therefore not independent. Model-1 may have higher weights in some combinations, and conversely, Model-2 might have higher weights in other combinations. Consequently, if both models are rewarded in the same set of weights, it is very likely that each model receives a reduced weight since both models are providing similar information to the model average. See Supplementary Section 2 of Massoud et al., (2020) for additional details on how dependence is inferred with the BMA method. For additional details on how the BMA method is applied in this context, see Massoud et al., (2019, 2020) and Wootten et al., (2020, 2023).

### Supplementary information

Peer review

Supplementary Information (.pdf)

## Data Availability

The code used to generate results for this study can be found on GitHub at https://github.com/EliasMassoud1/BMA_ECS.

## References

[1] Eyring V (2016). Overview of the Coupled Model Intercomparison Project Phase 6 (CMIP6) experimental design and organization. Geosci. Model Dev..

[2] Zelinka MD (2020). Causes of higher climate sensitivity in CMIP6 models. Geophys. Res. Lett..

[3] Nijsse FJMM, Cox PM, Williamson MS (2020). Emergent constraints on transient climate response (TCR) and equilibrium climate sensitivity (ECS) from historical warming in CMIP5 and CMIP6 models. Earth Syst. Dynam..

[4] Tokarska KB (2020). Past warming trend constrains future warming in CMIP6 models. Sci. Adv..

[5] Liang Y, Gillett NP, Monahan AH (2020). Climate model projections of 21st century global warming constrained using the observed warming trend. Geophys. Res. Lett..

[6] Zhu J, Poulsen CJ, Otto-Bliesner BL (2020). High climate sensitivity in CMIP6 model not supported by paleoclimate. Nat. Clim. Change.

[7] Sherwood SC (2020). An assessment of Earth’s climate sensitivity using multiple lines of evidence. Rev. Geophys..

[8] Ribes A, Qasmi S, Gillett NP (2021). Making climate projections conditional on historical observations. Sci. Adv..

[9] Lee, J. Y., et al, 2021: Future global climate: scenario-based projections and near term information. In Climate change 2021: the physical science basis. Contribution of Working Group I to the Sixth Assessment Report of the Intergovernmental Panel on Climate Change (eds. Masson-Delmotte, V., et al.) pp. 553–672 (Cambridge University Press, Cambridge, United Kingdom and New York, NY, USA).

[10] Hausfather Z, Marvel K, Schmidt GA, Nielsen-Gammon JW, Zelinka M (2022). Climate simulations: recognize the ‘hot model’ problem. Nature.

[11] Asenjan, M. R., Brissette, F., Martel, J.-L., & Arsenault, R. The Dilemma of Including ‘Hot’ Models in Climate Impact Studies: A Hydrological Study, Hydrol. Earth Syst. Sci. Discuss. (preprint), 10.5194/hess-2023-47, in review, 2023.

[12] Tebaldi C (2021). Extreme sea levels at different global warming levels. Nat. Clim. Change.

[13] Knutti R (2010). The end of model democracy?. Clim. Change.

[14] Bloch-Johnson J, Rugenstein M, Gregory J, Cael BB, Andrews T (2022). Climate impact assessments should not discount ‘hot’ models. Nature.

[15] Draper D (1995). Assessment and propagation of model uncertainty. J. R. Stat. Soc. Ser. B.

[16] Bhat KS, Haran M, Terando A, Keller K (2011). Climate projections using bayesian model averaging and space–time dependence. J. Agric. Biol. Environ. Stat..

[17] Massoud EC, Espinoza V, Guan B, Waliser DE (2019). Global climate model ensemble approaches for future projections of atmospheric rivers. Earth’s Future.

[18] Massoud EC, Lee H, Gibson PB, Loikith P, Waliser DE (2020). Bayesian model averaging of climate model projections constrained by precipitation observations over the contiguous United States. J. Hydrometeorol..

[19] Wootten AM, Massoud EC, Sengupta A, Waliser DE, Lee H (2020). The effect of statistical downscaling on the weighting of multi-model ensembles of precipitation. Climate.

[20] Wootten AM, Massoud EC, Waliser DE, Lee H (2023). Assessing sensitivities of climate model weighting to multiple methods, variables, and domains in the south-central United States. Earth Syst. Dynam..

[21] Sanderson, B. M., Wehner, M., and Knutti, R. Skill and independence weighting for multi-model assessments. *Geosci. Model Dev*, 2379–2395, 10.5194/gmd-2016-285 (2017).

[22] Min, S. K., and Hense A. A Bayesian approach to climate model evaluation and multi‐model averaging with an application to global mean surface temperatures from IPCC AR4 coupled climate models. *Geophys. Res. Lett.* 33.8 10.1029/2006GL025779 (2006).

[23] Tebaldi C, Knutti R (2007). The use of the multi-model ensemble in probabilistic climate projections. Philos. Trans. R. Soc. A.

[24] Jun M, Knutti R, Nychka DW (2008). Spatial analysis to quantify numerical model bias and dependence. J. Am. Stat. Assoc..

[25] Weigel AP, Knutti R, Liniger MA, Appenzeller C (2010). Risks of model weighting in multimodel climate projections. J. Clim..

[26] Klocke D, Pincus R, Quaas J (2011). On constraining estimates of climate sensitivity with present-day observations through model weighting. J. Clim..

[27] DelSole T, Yang X, Tippett MK (2013). Is unequal weighting significantly better than equal weighting for multi-model forecasting?. Q. J. R. Meteorol. Soc..

[28] Merrifield AL, Brunner L, Lorenz R, Medhaug I, Knutti R (2020). An investigation of weighting schemes suitable for incorporating large ensembles into multi-model ensembles. Earth Syst. Dynam..

[29] Brunner L (2020). Reduced global warming from CMIP6 projections when weighting models by performance and independence. Earth Syst. Dynam..

[30] Peña M, van den Dool H (2008). Consolidation of multimodel forecasts by ridge regression: application to Pacific sea surface temperature. J. Clim..

[31] Min SK, Simonis D, Hense A (2007). Probabilistic climate change predictions applying Bayesian model averaging. Philos. Trans. R. S. A: Math. Phys. Eng. Sci..

[32] Berliner LM, Kim Y (2008). Bayesian design and analysis for superensemble-based climate forecasting. J. Clim..

[33] Aldrin M (2012). Bayesian estimation of climate sensitivity based on a simple climate model fitted to observations of hemispheric temperatures and global ocean heat content. Environmetrics.

[34] Olson R, Fan Y, Evans JP (2016). A simple method for Bayesian model averaging of regional climate model projections: application to southeast Australian temperatures. Geophys. Res. Lett..

[35] Jonko A, Urban NM, Nadiga B (2018). Towards Bayesian hierarchical inference of equilibrium climate sensitivity from a combination of CMIP5 climate models and observational data. Clim. Change.

[36] Schillinger M (2022). Separating internal and externally forced contributions to global temperature variability using a Bayesian stochastic energy balance framework. Chaos: Interdiscip. J. Nonlinear Sci..

[37] Knutti R (2017). A climate model projection weighting scheme accounting for performance and interdependence. Geophys. Res. Lett..

[38] Sanderson BM, Knutti R, Caldwell P (2015). Addressing interdependency in a multimodel ensemble by interpolation of model properties. J, Clim..

[39] Sanderson, B. M. and Wehner, M. F. Model weighting strategy. In: *Climate Science Special Report: Fourth National Climate Assessment*, Vol. I (eds. Wuebbles, D. J., et al.). pp. 436–442 (U.S. Global Change Research Program, Washington, DC, USA, 2017).

[40] IPCC, 2022: Climate Change 2022: Impacts, Adaptation, and Vulnerability. Contribution of Working Group II to the Sixth Assessment Report of the Intergovernmental Panel on Climate Change [eds H.-O. Pörtner, et al.) (Cambridge University Press. In Press).

[41] Scafetta N (2021). Testing the CMIP6 GCM simulations versus surface temperature records from 1980–1990 to 2011–2021: high ECS Is not supported. Climate.

[42] Gonzalez Cruz M, Hernandez EA, Uddameri V (2020). Climatic influences on agricultural drought risks using semiparametric kernel density estimation. Water.

[43] Jeffreys H (1946). An invariant form for the prior probability in estimation problems. Proc. R. Soc. London. Ser. A, Math. Phys. Sci..

