# Supplementary material for: Bayesian weighting of climate models based on climate sensitivity
Source: Commun Earth Environ. 2023 Oct 20;4(1):365. doi: 10.1038/s43247-023-01009-8 (PMC11041668; doi:10.1038/s43247-023-01009-8)
Supplement: Supplementary file 2 — Supplementary Information (.pdf) [file 43247_2023_1009_MOESM2_ESM.pdf]

Supplementary Material:

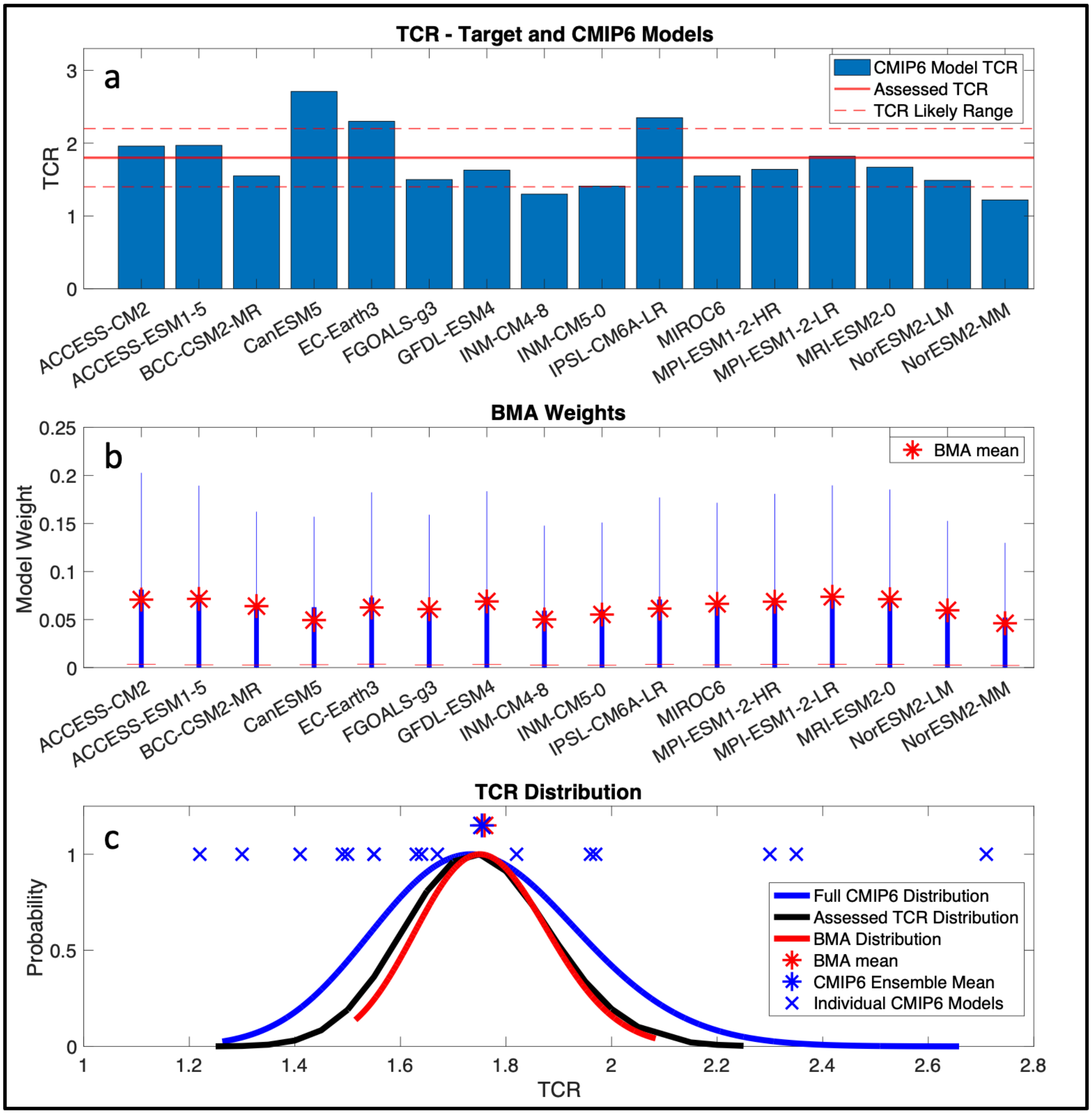

Fig. S1: Model weighting using TCR as the main fitting target.
a Transient Climate Response (TCR) for 16 Earth System Models (ESMs) from the CMIP6 archive. The red line here depicts the IPCC assessed central value estimate of TCR, which is 1.8°C (dashed red lines show the upper and lower bounds of the assessed TCR distribution). **b** BMA posterior distributions (blue box-and-whisker plots) of the model weights after using the assessed TCR distribution as a fitting metric, with the mean BMA weights shown with the red stars. **c** The TCR value from each CMIP6 model (blue x) with the distribution from the raw CMIP6 ensemble estimated from Monte Carlo sampling of the model weight space (blue curve), the target assessed TCR distribution (black curve), and the final BMA estimated posterior distribution of TCR (red curve).

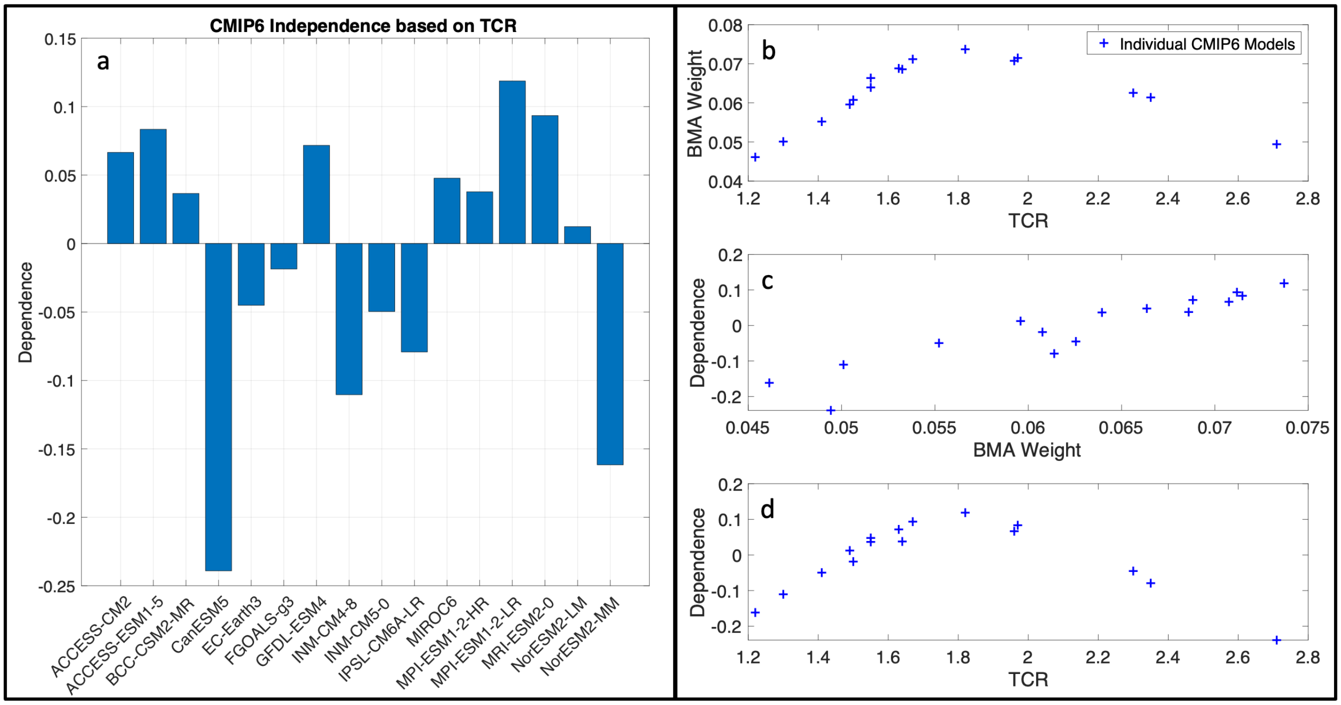

Fig. S2: Relations between model weight, independence, and TCR scores.
a This plot shows the bar graph of the model dependence scores estimated from the BMA posterior distributions when using the TCR as a fitting metric. A higher (more positive) value indicates a model with higher dependence on other models (i.e., a less independent model), while a lower (more negative) value indicates a model with less dependence (i.e., a more independent model). **b-d** These panels show scatter plots of each individual CMIP6 model and the relationship between **b** the BMA weight and the corresponding TCR value, **c** the dependence score and corresponding BMA weight, and **d** the dependence score and corresponding TCR value. This figure highlights how models that are 'too hot' have lower BMA weights and dependence scores, and this decrease in weight drops almost linearly with increasing TCR value. For reference, all models would have a weight of 1/16 or 0.0625 if equal weights were applied.

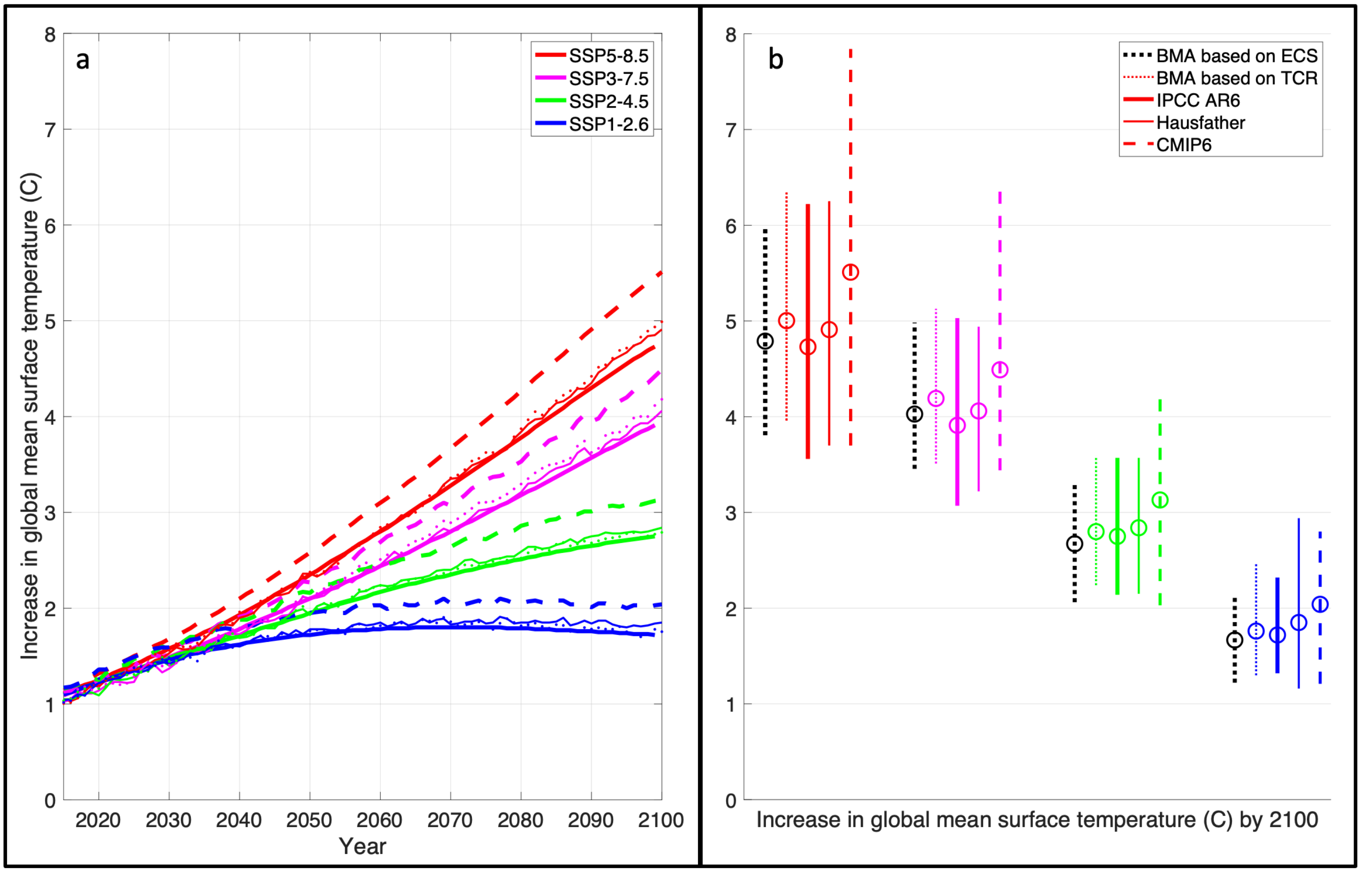

Fig. S3: Future projections of global mean surface temperature based on TCR.
a Increase in global mean surface temperature (°C) for the different SSP scenarios considered and the different model averaging methods used. Dashed lines are the raw CMIP6 mean, light solid lines are from Hausfather et al., dark solid lines are the AR6 assessed warming levels, and colored dotted lines are the results produced in this paper from the BMA method when using TCR as a fitting metric. **b** Increase in global mean surface temperature by the year 2100 and the uncertainty ranges of this estimate for each SSP scenario and each model averaging method considered here. The results from the BMA-ECS analysis (black dotted line) from the main text are also included in this panel for comparison with the BMA-TCR results. Results shown here have no temporal filtering. The BMA uncertainty bar plotted here is the top 95% of the full posterior distribution of model weights.
