# Supplementary material for: Bayesian weighting of climate models based on climate sensitivity
Source: Commun Earth Environ. 2023 Oct 20;4(1):365. doi: 10.1038/s43247-023-01009-8 (PMC11041668; doi:10.1038/s43247-023-01009-8)
Supplement: Supplementary file 1 — Peer review [file 43247_2023_1009_MOESM1_ESM.pdf]

nature portfolio

# Peer Review File

## Bayesian weighting of climate models based on climate sensitivity

**Open Access** This file is licensed under a Creative Commons Attribution 4.0 International License, which permits use, sharing, adaptation, distribution and reproduction in any medium or format, as long as you give appropriate credit to the original author(s) and the source, provide a link to the Creative Commons license, and indicate if changes were made. In the cases where the authors are anonymous, such as is the case for the reports of anonymous peer reviewers, author attribution should be to 'Anonymous Referee' followed by a clear attribution to the source work. The images or other third party material in this file are included in the article's Creative Commons license, unless indicated otherwise in a credit line to the material. If material is not included in the article's Creative Commons license and your intended use is not permitted by statutory regulation or exceeds the permitted use, you will need to obtain permission directly from the copyright holder. To view a copy of this license, visit http://creativecommons.org/licenses/by/4.0/.

Web links to the author's journal account have been redacted from the decision letters as indicated to maintain confidentiality.

Dear Dr Massoud,

Your manuscript titled "Bayesian weighting of climate models based on climate sensitivity" has now been seen by three reviewers, and I include their comments at the end of this message. They find your work of interest, but some important points are raised. We are interested in the possibility of publishing your study in Communications Earth & Environment, but would like to consider your responses to these concerns and assess a revised manuscript before we make a final decision on publication.

We therefore invite you to revise and resubmit your manuscript, along with a point-by-point response that takes into account the points raised.
Specifically, for publication in Communications Earth & Environment to be appropriate, we will need you to
* discuss previous research on the topic more extensively, and compare alternatives to your own method in more detail
* provide sufficient detail on your methods to allow an independent researcher to reproduce your study
We also strongly endorse reviewer 3's request that you make the code available.

Please highlight all changes in the manuscript text file.

We are committed to providing a fair and constructive peer-review process. Please don't hesitate to contact us if you wish to discuss the revision in more detail.

Please use the following link to submit your revised manuscript, point-by-point response to the referees' comments (which should be in a separate document to any cover letter) and the completed checklist:
[Link redacted]

** This url links to your confidential home page and associated information about manuscripts you may have submitted or be reviewing for us. If you wish to forward this email to co-authors, please delete the link to your homepage first **

We hope to receive your revised paper within six weeks; please let us know if you aren't able to submit it within this time so that we can discuss how best to proceed. If we don't hear from you, and the revision process takes significantly longer, we may close your file. In this event, we will still be happy to reconsider your paper at a later date, as long as nothing similar has been accepted for publication at Communications Earth & Environment or published elsewhere in the meantime.

We understand that due to the current global situation, the time required for revision may be longer than usual. We would appreciate it if you could keep us informed about an estimated timescale for resubmission, to facilitate our planning. Of course, if you are unable to estimate, we are happy to accommodate necessary extensions nevertheless.

Please do not hesitate to contact me if you have any questions or would like to discuss these revisions further. We look forward to seeing the revised manuscript and thank you for the opportunity to review your work.

Best regards,

Heike Langenberg, PhD
Chief Editor
Communications Earth & Environment

On Twitter: @CommsEarth

EDITORIAL POLICIES AND FORMATTING

We ask that you ensure your manuscript complies with our editorial policies. Please ensure that the following formatting requirements are met, and any checklist relevant to your research is completed and uploaded as a Related Manuscript file type with the revised article.

Editorial Policy: <a href="https://www.nature.com/documents/nr-editorial-policy-checklist.pdf">Policy requirements </a> (Download the link to your computer as a PDF.)

Furthermore, please align your manuscript with our format requirements, which are summarized on the following checklist:
<a href="https://www.nature.com/documents/commsj-phys-style-formatting-checklist-article.pdf">Communications Earth & Environment formatting checklist</a>

and also in our style and formatting guide <a href="https://www.nature.com/documents/commsj-phys-style-formatting-guide-accept.pdf">Communications Earth & Environment formatting guide</a>
.

*** DATA: Communications Earth & Environment endorses the principles of the Enabling FAIR data project (http://www.copdess.org/enabling-fair-data-project/ ). We ask authors to make the data that support their conclusions available in permanent, publically accessible data repositories. (Please contact the editor if you are unable to make your data available).

All Communications Earth & Environment manuscripts must include a section titled "Data Availability" at the end of the Methods section or main text (if no Methods). More information on this policy, is available at <a href="http://www.nature.com/authors/policies/data/data-availability-statements-data-citations.pdf">http://www.nature.com/authors/policies/data/data-availability-statements-data-citations.pdf</a>.

In particular, the Data availability statement should include:
- Unique identifiers (such as DOIs and hyperlinks for datasets in public repositories)
- Accession codes where appropriate
- If applicable, a statement regarding data available with restrictions
- If a dataset has a Digital Object Identifier (DOI) as its unique identifier, we strongly encourage including this in the Reference list and citing the dataset in the Data Availability Statement.

DATA SOURCES: All new data associated with the paper should be placed in a persistent repository where they can be freely and enduringly accessed. We recommend submitting the data to discipline-specific, community-recognized repositories, where possible and a list of recommended repositories is provided at <a href="http://www.nature.com/sdata/policies/repositories">http://www.nature.com/sdata/policies/repositories</a>.

If a community resource is unavailable, data can be submitted to generalist repositories such as <a href="https://figshare.com/">figshare</a> or <a href="http://datadryad.org/">Dryad Digital Repository</a>. Please provide a unique identifier for the data (for example a DOI or a permanent URL) in the data availability statement, if possible. If the repository does not provide identifiers, we encourage authors to supply the search terms that will return the data. For data that have been obtained from publically available sources, please provide a URL and the specific data product name in the data availability statement. Data with a DOI should be further cited in the methods reference section.

Please refer to our data policies at <a href="http://www.nature.com/authors/policies/availability.html">http://www.nature.com/authors/policies/availability.html</a>.

REVIEWER COMMENTS:

Reviewer #1 (Remarks to the Author):

Overview
This manuscript shows results of a Bayesian Model Averaging method to constrain future climate projections, so as to reduce the weight of 'too hot' climate models with high climate sensitivities. The authors contrast their method against model culling, which fully excludes too hot models from the ensemble, and approaches including multiple lines of evidence used by the IPCC WG1. They make the argument that their method is simpler than the latter, while still maintaining the full ensemble, in contrast to the former.

Overall, the manuscript is well-written, concise and clear. However, it lacks information on the Bayesian Model Averaging method used that would allow the reader to understand what was actually done, or to reproduce the results. In addition, the authors do not discuss the large body of previous work on model weighting, including using various Bayesian approaches, and some of the authors' previous work. The reference list of only 21 previous studies seems to indicate that this is the first such attempt, and that is not accurate.

The results themselves are not particularly novel, as they basically reproduce ensemble mean surface temperature projections and ECS estimates provided by AR6 and Hausfather et al. What could be novel about this work would be the method to average models. However, no information about it is provided, so this is difficult to assess. To improve the manuscript, I recommend that the authors focus on (1) explaining the Bayesian method used in some detail, and (2) putting it into perspective by adequately discussing the large body of work that has been done in this area.

Specific comments
L24-26 I would recommend mentioning some specific results in the final sentence of the abstract

L40 I would replace "proffered" with "proposed"

L40-51 This is really not an adequate discussion of previous work. See the references provided below, and consider expanding this to more accurately represent the body of previous work on this topic.

L73 the "best estimate" ECS value of 3C is used here as a realistic/true ECS value. I feel that this is an overstatement of confidence. It would be good to acknowledge the uncertainties associated with that. Many previous studies employ observational data as a constraint in a Bayesian framework, and I think the authors are attempting to do the same with this "true ECS", however, they are fundamentally different things, and this needs to be acknowledged.

L109-116 This paragraph talks about obtaining information about model dependence/independence from the Bayesian posterior, but again, the authors do not explain how this is done. Please provide more information.

L155-157 This sentence is confusing and vague. What does "properly" mean? Are the "methods shown here" including Hausfather et al? The sentence implies that it would be methods the authors propose. Maybe reword as follows: "When the 'hot model' problem is addressed, either by down-weighting, or culling, future global mean temperatures are lower, and increases less exaggerated."

Although I am not 100% sold on using 'exaggerated', as it suggests that we know these models are wrong, and if they were, why wouldn’t we just exclude them from the ensemble?

L161-164 The reasoning behind the observation made here needs more explanation. It’s unclear what is meant by elimination of information of how these models simulate climate in a low emission scenario.

L 250-251 This reference is incomplete

Figure 1: Please label the 3 panels with A), B), and C), as referenced in the caption. In A), the red line, does not actually show up. In B) Consider using a circle for the legend, to correspond to the symbols in the figure. Also explain what the blue bars mean. In C) the black and navy circles are basically indistinguishable, please use other colors.

Figure 2: Same as figure 1, please label the panels. The line types in A) don't easily correspond to the ones in panel B). In A) three lines are dark and one is light, vs in B) two lines are dark and two light.

Some previous work worth discussing in a revised version of the manuscript, in reverse chronological order

Schillinger, Maybritt, et al. "Separating internal and externally forced contributions to global temperature variability using a Bayesian stochastic energy balance framework." Chaos: An Interdisciplinary Journal of Nonlinear Science 32.11 (2022): 113146.

Massoud, E. C., et al. "Bayesian model averaging of climate model projections constrained by precipitation observations over the contiguous United States." Journal of Hydrometeorology 21.10 (2020): 2401-2418.

Brunner, Lukas, et al. "Reduced global warming from CMIP6 projections when weighting models by performance and independence." Earth System Dynamics 11.4 (2020): 995-1012.

Merrifield, Anna Louise, et al. "An investigation of weighting schemes suitable for incorporating large ensembles into multi-model ensembles." Earth System Dynamics 11.3 (2020): 807-834.

Jonko, A., Urban, N. M., & Nadiga, B. (2018). Towards Bayesian hierarchical inference of equilibrium climate sensitivity from a combination of CMIP5 climate models and observational data. Climatic Change, 149(2), 247-260.

Lorenz, R., Herger, N., Sedláček, J., Eyring, V., Fischer, E. M., & Knutti, R. (2018). Prospects and caveats of weighting climate models for summer maximum temperature projections over North America. Journal of Geophysical Research: Atmospheres, 123(9), 4509-4526.

Knutti, Reto, et al. "A climate model projection weighting scheme accounting for performance and interdependence." Geophysical Research Letters 44.4 (2017): 1909-1918.

Olson, Roman, Yanan Fan, and Jason P. Evans. "A simple method for Bayesian model averaging of regional climate model projections: Application to southeast Australian temperatures." Geophysical Research Letters 43.14 (2016): 7661-7669.

DelSole T, Yang X, Tippett MK (2013) Is unequal weighting significantly better than equal weighting for multi-model forecasting? Q J R Meteorol Soc 139:176–183

Aldrin M, Holden M, Guttorp P et al (2012) Bayesian estimation of climate sensitivity based on a simple climate model fitted to observations of hemispheric temperatures and global ocean heat

content. Environmetrics 23:253–271

Klocke D, Pincus R, Quaas J (2011) On constraining estimates of climate sensitivity with present-day observations through model weighting. J Clim 24:6092–6099

Bhat, K. Sham, et al. "Climate projections using Bayesian model averaging and space–time dependence." Journal of Agricultural, Biological, and Environmental Statistics 16.4 (2011): 606-628.

Knutti R, Furrer R, Tebaldi C et al (2010) Challenges in Combining Projections from Multiple Climate Models. J Clim 23:2739–2758

Räisänen J, Ruokolainen L, Ylhäisi J (2010) Weighting of model results for improving best estimates of climate change. Clim Dyn 35:407–422

Weigel, A. P., Knutti, R., Liniger, M. A., & Appenzeller, C. (2010). Risks of model weighting in multimodel climate projections. Journal of Climate, 23(15), 4175-4191.

Jun M, Knutti R, Nychka DW (2008) Spatial Analysis to Quantify Numerical Model Bias and Dependence. J Am Stat Assoc 103:934–947

Berliner LM, Kim Y (2008) Bayesian Design and Analysis for Superensemble-Based Climate Forecasting. J Clim 21:1891–1910

Tebaldi C, Knutti R (2007) The use of the multi-model ensemble in probabilistic climate projections. Philos Trans R Soc A 365:2053–2075

Min, Seung-Ki, Daniel Simonis, and Andreas Hense. "Probabilistic climate change predictions applying Bayesian model averaging." Philosophical Transactions of the Royal Society A: Mathematical, Physical and Engineering Sciences 365.1857 (2007): 2103-2116.

Min, Seung-Ki, and Andreas Hense. "A Bayesian approach to climate model evaluation and multi-model averaging with an application to global mean surface temperatures from IPCC AR4 coupled climate models." Geophysical Research Letters 33.8 (2006).

Reviewer #2 (Remarks to the Author):

See attached review

Reviewer #3 (Remarks to the Author):

Review of "Bayesian weighting of climate models based on climate sensitivity" by E. C. Massoud and co-authors.

In this study the authors propose a method to weigh climate models based on their equilibrium climate sensitivity (ECS) in order to make projections. This is a welcome initiative relative to previous suggestions to use some kind of performance metric in how well a model represents the present-day mean climate. As the authors explain, there are other alternatives, for instance IPCC AR6 used emulators with prescribed ECS and global warming levels, whereas Hausfather et al. suggested rejecting som models.

Overall, I have not much to say about this paper, it has a clear goal and is fairly well written. I do not share the authors enthusiasm for their method, personally I prefer the IPCC approach for its simplicity. I did not find the Bayesian approach with 10000 samples particular transparent, but then again I am biased. After addressing the below minor points, I recommend publication.

---

The IPCC AR6 assessed ECS likely range is 2.5-4.0 K, but does not provide a probability distribution. The likely range means greater than 66 percent probability to be within, and furthermore, the range is not symmetric about the best estimate of 3.0. Also the IPCC AR6 state they work with 0.5 K precision. Yet, judging from the 'Expected truth' in Figure 1c, it seems the authors assumed a symmetric gaussian distribution which appears to be much tighter than what can be motivated by the IPCC AR6 assessment. I suggest the authors change to using a target distribution which better represents the IPCC AR6 assessment. Also, they should document their choice.

Furthermore, for transparence, I recommend the program that the authors use to do their calculations is made publicly available.

**Overview**

This manuscript shows results of a Bayesian Model Averaging method to constrain future climate projections, so as to reduce the weight of ‘too hot’ climate models with high climate sensitivities. The authors contrast their method against model culling, which fully excludes too hot models from the ensemble, and approaches including multiple lines of evidence used by the IPCC WG1. They make the argument that their method is simpler than the latter, while still maintaining the full ensemble, in contrast to the former.

Overall, the manuscript is well-written, concise and clear. However, it lacks information on the Bayesian Model Averaging method used that would allow the reader to understand what was actually done, or to reproduce the results. In addition, the authors do not discuss the large body of previous work on model weighting, including using various Bayesian approaches, and some of the authors’ previous work. The reference list of only 21 previous studies seems to indicate that this is the first such attempt, and that is not accurate.

The results themselves are not particularly novel, as they basically reproduce ensemble mean surface temperature projections and ECS estimates provided by AR6 and Hausfather et al. What could be novel about this work would be the method to average models. However, no information about it is provided, so this is difficult to assess. To improve the manuscript, I recommend that the authors focus on (1) explaining the Bayesian method used in some detail, and (2) putting it into perspective by adequately discussing the large body of work that has been done in this area.

**Specific comments**

L24-26 I would recommend mentioning some specific results in the final sentence of the abstract

L40 I would replace “proffered” with “proposed”

L40-51 This is really not an adequate discussion of previous work. See the references provided below, and consider expanding this to more accurately represent the body of previous work on this topic.

L73 the “best estimate” ECS value of 3C is used here as a realistic/true ECS value. I feel that this is an overstatement of confidence. It would be good to acknowledge the uncertainties associated with that. Many previous studies employ observational data as a constraint in a Bayesian framework, and I think the authors are attempting to do the same with this “true ECS”, however, they are fundamentally different things, and this needs to be acknowledged.

L109-116 This paragraph talks about obtaining information about model dependence/independence from the Bayesian posterior, but again, the authors do not explain how this is done. Please provide more information.

L155-157 This sentence is confusing and vague. What does "properly" mean? Are the "methods shown here" including Hausfather et al? The sentence implies that it would be methods the authors propose. Maybe reword as follows: "When the 'hot model' problem is addressed, either by down-weighting, or culling, future global mean temperatures are lower, and increases less exaggerated."

Although I am not 100% sold on using 'exaggerated', as it suggests that we know these models are wrong, and if they were, why wouldn’t we just exclude them from the ensemble?

L161-164 The reasoning behind the observation made here needs more explanation. It's unclear what is meant by elimination of information of how these models simulate climate in a low emission scenario.

L 250-251 This reference is incomplete

Figure 1: Please label the 3 panels with A), B), and C), as referenced in the caption. In A), the red line, does not actually show up. In B) Consider using a circle for the legend, to correspond to the symbols in the figure. Also explain what the blue bars mean. In C) the black and navy circles are basically indistinguishable, please use other colors.

Figure 2: Same as figure 1, please label the panels. The line types in A) don't easily correspond to the ones in panel B). In A) three lines are dark and one is light, vs in B) two lines are dark and two light.

**Some previous work worth discussing in a revised version of the manuscript, in reverse chronological order**


Schillinger, Maybritt, et al. "Separating internal and externally forced contributions to global temperature variability using a Bayesian stochastic energy balance framework." Chaos: An Interdisciplinary Journal of Nonlinear Science 32.11 (2022): 113146.

Massoud, E. C., et al. "Bayesian model averaging of climate model projections constrained by precipitation observations over the contiguous United States." Journal of Hydrometeorology 21.10 (2020): 2401-2418.

Brunner, Lukas, et al. "Reduced global warming from CMIP6 projections when weighting models by performance and independence." Earth System Dynamics 11.4 (2020): 995-1012.

Merrifield, Anna Louise, et al. "An investigation of weighting schemes suitable for incorporating large ensembles into multi-model ensembles." Earth System Dynamics 11.3 (2020): 807-834.

Jonko, A., Urban, N. M., & Nadiga, B. (2018). Towards Bayesian hierarchical inference of equilibrium climate sensitivity from a combination of CMIP5 climate models and observational data. Climatic Change, 149(2), 247-260.

Lorenz, R., Herger, N., Sedláček, J., Eyring, V., Fischer, E. M., & Knutti, R. (2018). Prospects and caveats of weighting climate models for summer maximum temperature projections over North America. Journal of Geophysical Research: Atmospheres, 123(9), 4509-4526.

Knutti, Reto, et al. "A climate model projection weighting scheme accounting for performance and interdependence." Geophysical Research Letters 44.4 (2017): 1909-1918.

Olson, Roman, Yanan Fan, and Jason P. Evans. "A simple method for Bayesian model averaging of regional climate model projections: Application to southeast Australian temperatures." Geophysical Research Letters 43.14 (2016): 7661-7669.

DelSole T, Yang X, Tippett MK (2013) Is unequal weighting significantly better than equal weighting for multi-model forecasting? Q J R Meteorol Soc 139:176–183

Aldrin M, Holden M, Guttorp P et al (2012) Bayesian estimation of climate sensitivity based on a simple climate model fitted to observations of hemispheric temperatures and global ocean heat content. Environmetrics 23:253–271

Klocke D, Pincus R, Quaas J (2011) On constraining estimates of climate sensitivity with present-day observations through model weighting. J Clim 24:6092–6099

Bhat, K. Sham, et al. "Climate projections using Bayesian model averaging and space–time dependence." Journal of Agricultural, Biological, and Environmental Statistics 16.4 (2011): 606-628.

Knutti R, Furrer R, Tebaldi C et al (2010) Challenges in Combining Projections from Multiple Climate Models. J Clim 23:2739–2758

Räisänen J, Ruokolainen L, Ylhäisi J (2010) Weighting of model results for improving best estimates of climate change. Clim Dyn 35:407–422

Weigel, A. P., Knutti, R., Liniger, M. A., & Appenzeller, C. (2010). Risks of model weighting in multimodel climate projections. Journal of Climate, 23(15), 4175-4191.

Jun M, Knutti R, Nychka DW (2008) Spatial Analysis to Quantify Numerical Model Bias and Dependence. J Am Stat Assoc 103:934–947

Berliner LM, Kim Y (2008) Bayesian Design and Analysis for Superensemble-Based Climate Forecasting. J Clim 21:1891–1910

Tebaldi C, Knutti R (2007) The use of the multi-model ensemble in probabilistic climate projections. Philos Trans R Soc A 365:2053–2075

Min, Seung-Ki, Daniel Simonis, and Andreas Hense. "Probabilistic climate change predictions applying Bayesian model averaging." Philosophical Transactions of the Royal Society A: Mathematical, Physical and Engineering Sciences 365.1857 (2007): 2103-2116.

Min, Seung-Ki, and Andreas Hense. "A Bayesian approach to climate model evaluation and multi-model averaging with an application to global mean surface temperatures from IPCC AR4 coupled climate models." Geophysical Research Letters 33.8 (2006).

Review of Massaud et al., “Bayesian weighting of climate models…”

This paper proposes what it claims is a Bayesian model averaging scheme for producing a PDF of any projected quantity X, by proposing weights for the available models. Conceptually, I believe P(X) would then be given by the integral over the joint PDF of the weight on each model (i.e. by sampling the weight space one obtains a sample of predictions, whose sample distribution is the desired PDF).

The idea is interesting (not totally new). But this paper requires two important revisions, and checking one apparent problem, before it could be published:

1. The paper must explain its method! The authors simply say they are “applying BMA” and then show results—this is ridiculous, I have no idea what they’ve done. Also, they state that the process tends to pick models that are more independent, without saying what independence even means in this context (normally to decide model dependence one must compare some high-dimensional outputs to see which ones show more or less resemblance or cannot be obtained by linear combination of others). The paper is written as if there will be a separate Methods section, but I don’t see one (maybe it was omitted??)

2. The paper needs to articulate why this BMA method is any better than discarding “hot” models or what I’d call model truncation (Hausfather et al.), or any simpler weighting scheme such as assigning a weight to each model j proportional to the ratio of the desired P(ECS_j) to the local model sample density around ECS_j. Indeed, the best-estimate future warming trajectories the authors report are essentially identical with those obtained by the Hausfather approach. So why do we need this? The authors’ approach is harder to understand so there is a burden of proof here.
   What the authors aren’t saying is that the importance of their approach lies in the spread of the PDF of projected changes. Theirs is larger than those from model truncation, which underestimates the uncertainty of any projection. Following my “simple weighting” scheme above would avoid this problem, but then it wouldn’t be clear how to get a PDF, only a best estimate (the weighted average of the model estimates). The authors’ procedure, by producing a PDF of the weights, enables a PDF of predicted quantities. The authors need to highlight this point (including in the abstract) in order to make their contribution more valuable.

3. The performance of the method on TCR looks very poor (Fig. S3): the spread of the BMA distribution is way too narrow and we’d have been better off using the full CMIP6 distribution which is closer to “truth”. Why? This method should be able to replicate almost exactly the desired PDF, if I understand it correctly (whether this really carries over to projections is another story). I don’t see how this can work so poorly and it makes me think the authors have done something wrong. For that matter I don’t understand why there is a bias in the ECS results, even though small (Fig. 1, red curve slightly to left of black one). Indeed I am wondering if the authors are still using ECS to weight the models—it would in fact be a good test of the method to see how well it does on TCR if “trained” on ECS (or vice versa), but here it seems to be doing badly on TCR even though trained on TCR.

Minor comments

Most of the multi-panel figures (Figs. 1, 2, S2,S3,S5) refer to the panels by letter, but do not include letter labels on the figure.

Fig. 1: This is a nice figure but has a few minor glitches. The top panel legend has a second entry that says “Truth ECS” but doesn’t seem to refer to anything. The middle panel legend shows the mean with X but the caption says circle (figure has both—why?), and doesn’t explain what the thick and thin blue lines, or the red lines, indicate. In bottom panel, symbol shown for means is a circle but in the figure it is a circle with crosshairs.

Figs. S1, S2, S4 have grammatical mistakes in the captions.

Reviewer #1 (Remarks to the Author):

Overview
This manuscript shows results of a Bayesian Model Averaging method to constrain future climate projections, so as to reduce the weight of 'too hot' climate models with high climate sensitivities. The authors contrast their method against model culling, which fully excludes too hot models from the ensemble, and approaches including multiple lines of evidence used by the IPCC WG1. They make the argument that their method is simpler than the latter, while still maintaining the full ensemble, in contrast to the former.

Overall, the manuscript is well-written, concise and clear. However, it lacks information on the Bayesian Model Averaging method used that would allow the reader to understand what was actually done, or to reproduce the results. In addition, the authors do not discuss the large body of previous work on model weighting, including using various Bayesian approaches, and some of the authors' previous work. The reference list of only 21 previous studies seems to indicate that this is the first such attempt, and that is not accurate.

The results themselves are not particularly novel, as they basically reproduce ensemble mean surface temperature projections and ECS estimates provided by AR6 and Hausfather et al. What could be novel about this work would be the method to average models. However, no information about it is provided, so this is difficult to assess. To improve the manuscript, I recommend that the authors focus on (1) explaining the Bayesian method used in some detail, and (2) putting it into perspective by adequately discussing the large body of work that has been done in this area.

Author Comment: We thank the reviewer for the time spent reading and assessing our manuscript. We agree that additional information on the BMA method is needed, and that a more extensive literature review should be provided. To this end, we have provided a section describing the BMA method (Methods – Bayesian Model Averaging) starting on Line 243, and we have also provided a deeper list of relevant literature throughout the manuscript but especially in the newly added section (The Community's Evolution with Model weighting Strategies) starting on Line 91.

Specific comments
L24-26 I would recommend mentioning some specific results in the final sentence of the abstract

Author Comment: Thank you for this comment. We have included a couple of specific statements on the results in the abstract.

L40 I would replace "proffered" with "proposed"

Author Comment: Thank you for this comment. We fixed this.

L40-51 This is really not an adequate discussion of previous work. See the references provided below, and consider expanding this to more accurately represent the body of previous work on this topic.

Author Comment: We agree with this comment and have expanded the literature review throughout the manuscript but especially in the newly added section (The Community's Evolution with Model weighting Strategies) starting on Line 91.

L73 the "best estimate" ECS value of 3C is used here as a realistic/true ECS value. I feel that this is an overstatement of confidence. It would be good to acknowledge the uncertainties associated with that. Many previous studies employ observational data as a constraint in a Bayesian framework, and I think the authors are attempting to do the same with this "true ECS", however, they are fundamentally different things, and this needs to be acknowledged.

Author Comment: Thank you for this insight. We agree that the ECS value is different than normally applied observations in a BMA framework since ECS is not an observable quantity. The ECS value of 3C is the best guess, yet the uncertainty that we report and apply for this value ranges from 2.5C to 4.0C, as shown in the ECS distribution shown in Figure 1. Furthermore, the distribution that we use for ECS is a gamma distribution, with a peak near 3C and a range from 2.5C to 4.0C. This is all explained in more detail in the new section describing the methods (Methods – Bayesian Model Averaging) starting on Line 243.

L109-116 This paragraph talks about obtaining information about model dependence/independence from the Bayesian posterior, but again, the authors do not explain how this is done. Please provide more information.

Author Comment: Thank you for this comment. We have expanded the explanation of the method and how independence is estimated in the new section (Methods – Bayesian Model Averaging) starting on Line 243.

L155-157 This sentence is confusing and vague. What does "properly" mean? Are the "methods shown here" including Hausfather et al? The sentence implies that it would be methods the authors propose. Maybe reword as follows: "When the 'hot model' problem is addressed, either by down-weighting, or culling, future global mean temperatures are lower, and increases less exaggerated."
Although I am not 100% sold on using 'exaggerated', as it suggests that we know these models are wrong, and if they were, why wouldn't we just exclude them from the ensemble?

Author Comment: We agree that this statement can be misleading. We have taken the reviewer's suggestion and used the wording recommended here. This statement can now be seen on Line 193.

L161-164 The reasoning behind the observation made here needs more explanation.

It’s unclear what is meant by elimination of information of how these models simulate climate in a low emission scenario.

Author Comment: Thank you for this comment. The point we were trying to make here is an attempt to explain the results shown in Hausfather et al. In their paper, they show an increase in the spread of uncertainty in the SSP1-2.6 scenario compared to the original CMIP6 spread, which is counter-intuitive. The ideas we present in these sentences are simply trying to explain what is shown in Hausfather et al. We have edited this statement to make this clearer, on Line 199.

L 250-251 This reference is incomplete

Author Comment: Thank you. We have included the following in the citation:
Nature 605, 26-29 (2022), doi: https://doi.org/10.1038/d41586-022-01192-2.

Figure 1: Please label the 3 panels with A), B), and C), as referenced in the caption. In A), the red line, does not actually show up. In B) Consider using a circle for the legend, to correspond to the symbols in the figure. Also explain what the blue bars mean. In C) the black and navy circles are basically indistinguishable, please use other colors.

Author Comment: Thank you for this comment. The figure must have lost some of its formatting when being processed by the journal. We have fixed the figure according to these comments and will make sure that no information is lost in the next processing step.

Figure 2: Same as figure 1, please label the panels. The line types in A) don't easily correspond to the ones in panel B). In A) three lines are dark and one is light, vs in B) two lines are dark and two light.

Author Comment: Again, thank you for this comment. We have fixed the figure according to these comments.

Some previous work worth discussing in a revised version of the manuscript, in reverse chronological order

Schillinger, Maybritt, et al. "Separating internal and externally forced contributions to global temperature variability using a Bayesian stochastic energy balance framework." Chaos: An Interdisciplinary Journal of Nonlinear Science 32.11 (2022): 113146.

Massoud, E. C., et al. "Bayesian model averaging of climate model projections constrained by precipitation observations over the contiguous United States." Journal of Hydrometeorology 21.10 (2020): 2401-2418.

Brunner, Lukas, et al. "Reduced global warming from CMIP6 projections when weighting models by performance and independence." Earth System Dynamics 11.4 (2020): 995-1012.

Merrifield, Anna Louise, et al. "An investigation of weighting schemes suitable for incorporating large ensembles into multi-model ensembles." Earth System Dynamics 11.3 (2020): 807-834.

Jonko, A., Urban, N. M., & Nadiga, B. (2018). Towards Bayesian hierarchical inference of equilibrium climate sensitivity from a combination of CMIP5 climate models and observational data. Climatic Change, 149(2), 247-260.

Lorenz, R., Herger, N., Sedláček, J., Eyring, V., Fischer, E. M., & Knutti, R. (2018). Prospects and caveats of weighting climate models for summer maximum temperature projections over North America. Journal of Geophysical Research: Atmospheres, 123(9), 4509-4526.

Knutti, Reto, et al. "A climate model projection weighting scheme accounting for performance and interdependence." Geophysical Research Letters 44.4 (2017): 1909-1918.

Olson, Roman, Yanan Fan, and Jason P. Evans. "A simple method for Bayesian model averaging of regional climate model projections: Application to southeast Australian temperatures." Geophysical Research Letters 43.14 (2016): 7661-7669.

DelSole T, Yang X, Tippett MK (2013) Is unequal weighting significantly better than equal weighting for multi-model forecasting? Q J R Meteorol Soc 139:176–183

Aldrin M, Holden M, Guttorp P et al (2012) Bayesian estimation of climate sensitivity based on a simple climate model fitted to observations of hemispheric temperatures and global ocean heat content. Environmetrics 23:253–271

Klocke D, Pincus R, Quaas J (2011) On constraining estimates of climate sensitivity with present-day observations through model weighting. J Clim 24:6092–6099

Bhat, K. Sham, et al. "Climate projections using Bayesian model averaging and space–time dependence." Journal of Agricultural, Biological, and Environmental Statistics 16.4 (2011): 606-628.

Knutti R, Furrer R, Tebaldi C et al (2010) Challenges in Combining Projections from Multiple Climate Models. J Clim 23:2739–2758

Räisänen J, Ruokolainen L, Ylhäisi J (2010) Weighting of model results for improving best estimates of climate change. Clim Dyn 35:407–422

Weigel, A. P., Knutti, R., Liniger, M. A., & Appenzeller, C. (2010). Risks of model weighting in multimodel climate projections. Journal of Climate, 23(15), 4175-4191.

Jun M, Knutti R, Nychka DW (2008) Spatial Analysis to Quantify Numerical Model Bias

and Dependence. J Am Stat Assoc 103:934–947

Berliner LM, Kim Y (2008) Bayesian Design and Analysis for Superensemble-Based Climate Forecasting. J Clim 21:1891–1910

Tebaldi C, Knutti R (2007) The use of the multi-model ensemble in probabilistic climate projections. Philos Trans R Soc A 365:2053–2075

Min, Seung-Ki, Daniel Simonis, and Andreas Hense. "Probabilistic climate change predictions applying Bayesian model averaging." Philosophical Transactions of the Royal Society A: Mathematical, Physical and Engineering Sciences 365.1857 (2007): 2103-2116.

Min, Seung-Ki, and Andreas Hense. "A Bayesian approach to climate model evaluation and multi-model averaging with an application to global mean surface temperatures from IPCC AR4 coupled climate models." Geophysical Research Letters 33.8 (2006).

Author Comment: Thank you for these recommendations. We have included a broader literature review in the revised version of the manuscript. We hope you find the updated list of references more suitable for this paper.

Reviewer #2 (Remarks to the Author):
Review of Massaud et al., “Bayesian weighting of climate models…”

This paper proposes what it claims is a Bayesian model averaging scheme for producing a PDF of any projected quantity X, by proposing weights for the available models. Conceptually, I believe P(X) would then be given by the integral over the joint PDF of the weight on each model (i.e. by sampling the weight space one obtains a sample of predictions, whose sample distribution is the
desired PDF).

The idea is interesting (not totally new). But this paper requires two important revisions, and checking one apparent problem, before it could be published:

Author Comment: We thank the reviewer for the time spent reading and assessing our manuscript.

1. The paper must explain its method! The authors simply say they are “applying BMA” and then show results—this is ridiculous, I have no idea what they’ve done. Also, they state that the process tends to pick models that are more independent, without saying what independence even means in this context (normally to decide model dependence one must compare some high-dimensional outputs to see which ones show more or less resemblance or cannot be obtained by linear combination of others). The paper is written as if there will be a separate Methods section, but I don’t see one (maybe it was omitted??)

Author Comment: Thank you for this comment. We agree that additional information on the methods is needed, and we now provide a section describing the BMA method (Methods – Bayesian Model Averaging) starting on Line 243. Furthermore, the reviewer is correct, in that independence is normally measured by the difference in the model output space. However, for the BMA method presented here, the independence information is estimated during post-processing of the posterior distribution. This is now explained in more detail and we have expanded the explanation of how independence is estimated in the new section starting on Line 243.

2. The paper needs to articulate why this BMA method is any better than discarding “hot” models or what I’d call model truncation (Hausfather et al.), or any simpler weighting scheme such as assigning a weight to each model j proportional to the ratio of the desired P(ECS_j) to the local model sample density around ECS_j. Indeed, the best-estimate future warming trajectories the authors report are essentially identical with those obtained by the Hausfather approach. So why do we need this? The authors’ approach is harder to understand so there is a burden of proof here.
What the authors aren’t saying is that the importance of their approach lies in the spread of the PDF of projected changes. Theirs is larger than those from model truncation, which
underestimates the uncertainty of any projection. Following my “simple weighting” scheme above would avoid this problem, but then it wouldn’t be clear how to get a PDF,

only a best estimate (the weighted average of the model estimates). The authors' procedure, by producing a PDF of the weights, enables a PDF of predicted quantities. The authors need to highlight this point (including in the abstract) in order to make their contribution more valuable.

Author Comment: Thank you for this insightful comment. We agree that the inclusion of the points made here by the reviewer in the paper is warranted. To this end, we have included additional justification for the use of BMA in the newly added section (The Community's Evolution with Model weighting Strategies) starting on Line 91. Furthermore, we added statements on how the BMA method can beneficially provide the estimation and propagation of uncertainty. This information can be found in the abstract and throughout the manuscript (e.g. Lines 143, 160, 182). Lastly, the newly added section describing the BMA method (Methods – Bayesian Model Averaging) starting on Line 243 also provides details on how uncertainty is estimated. Furthermore, as explained in the text on Line 228, a principal motivation for our approach is not to just replicate the IPCC and Hausfather et al global mean surface temperature. Rather it is to find a way to build credible projections of other fields, such as precipitation, without losing information from the hot models, some which have demonstrated skill in replicating observed climatological patterns and magnitudes.

3. The performance of the method on TCR looks very poor (Fig. S3): the spread of the BMA distribution is way too narrow and we'd have been better off using the full CMIP6 distribution which is closer to "truth". Why? This method should be able to replicate almost exactly the desired PDF, if I understand it correctly (whether this really carries over to projections is another story). I don't see how this can work so poorly and it makes me think the authors have done something wrong. For that matter I don't understand why there is a bias in the ECS results, even though small (Fig. 1, red curve slightly to left of black one). Indeed I am wondering if the authors are still using ECS to weight the models—it would in fact be a good test of the method to see how well it does on TCR if "trained" on ECS (or vice versa), but here it seems to be doing badly on TCR even though trained on TCR.

Author Comment: Thank you for this comment. There are a couple of potential reasons why the BMA distributions do not exactly capture the ECS and TCR distributions. First, it is possible that this is the best BMA distribution that can be fitted against the ECS/TCR distributions given the set of 16 CMIP6 models to apply the BMA weighting on. Second, it is also possible that with additional sampling of the BMA weights (e.g., 1e7 or 1e9 samples compared to 1e5), that the exact posterior can be captured. Overall, we believe that the results shown in our paper serve the purpose of improving the ECS/TCR estimates since the BMA distributions are closer to the true ECS distributions compared to the original full CMIP6 distributions, and since the 'hot model' problem seems to be ameliorated with our BMA approach given the results in Figure 3.

Minor comments

Most of the multi-panel figures (Figs. 1, 2, S2,S3,S5) refer to the panels by letter, but do not include letter labels on the figure.

Author Comment: Thank you for this comment. The figure must have lost some of its formatting when being processed by the journal. We have fixed the figure according to these comments and will make sure that no information is lost in the next processing step.

Fig. 1: This is a nice figure but has a few minor glitches. The top panel legend has a second entry that says “Truth ECS” but doesn’t seem to refer to anything. The middle panel legend shows the mean with X but the caption says circle (figure has both—why?), and doesn’t explain what the thick and thin blue lines, or the red lines, indicate. In bottom panel, symbol shown for means is a circle but in the figure it is a circle with crosshairs.

Author Comment: Again, thank you for this comment. We have fixed the figure according to these comments.

Figs. S1, S2, S4 have grammatical mistakes in the captions.

Author Comment: Thank you for this comment. We have fixed the captions accordingly.

Reviewer #3 (Remarks to the Author):

Review of "Bayesian weighting of climate models based on climate sensitivity" by E. C. Massoud and co-authors.

In this study the authors propose a method to weigh climate models based on their equilibrium climate sensitivity (ECS) in order to make projections. This is a welcome initiative relative to previous suggestions to use some kind of performance metric in how well a model represents the present-day mean climate. As the authors explain, there are other alternatives, for instance IPCC AR6 used emulators with prescribed ECS and global warming levels, whereas Hausfather et al. suggested rejecting som models.

Overall, I have not much to say about this paper, it has a clear goal and is fairly well written. I do not share the authors enthusiasm for their method, personally I prefer the IPCC approach for its simplicity. I did not find the Bayesian approach with 10000 samples particular transparent, but then again I am biased. After addressing the below minor points, I recommend publication.

Author Comment: We thank the reviewer for the time spent reading and assessing our manuscript.

---

The IPCC AR6 assessed ECS likely range is 2.5-4.0 K, but does not provide a probability distribution. The likely range means greater than 66 percent probability to be within, and furthermore, the range is not symmetric about the best estimate of 3.0. Also the IPCC AR6 state they work with 0.5 K precision. Yet, judging from the 'Expected truth' in Figure 1c, it seems the authors assumed a symmetric gaussian distribution which appears to be much tighter than what can be motivated by the IPCC AR6 assessment. I suggest the authors change to using a target distribution which better represents the IPCC AR6 assessment. Also, they should document their choice.

Author Comment: Thank you for this insightful comment. To clarify, we do not use a gaussian distribution for ECS/TCR but rather we use a gamma distribution (2.5-4 C for ECS, and 1.4-2.2 C for TCR). The figure might make the distributions seem gaussian, but they are not applied that way. More information on the choice of fitting distributions and on the method in general have been added in the new section describing the BMA method (Methods – Bayesian Model Averaging) starting on Line 243.

Furthermore, for transparence, I recommend the program that the authors use to do their calculations is made publicly available.

Author Comment: Thank you for this comment. We agree, and our code to run this analysis is now available on GitHub at the following link [https://github.com/EliasMassoud1/BMA_ECS]. This information has been added to the manuscript in the “Code Availability” section.

Dear Dr Massoud,

Your revised manuscript titled "Bayesian weighting of climate models based on climate sensitivity" has now been seen by the original reviewers 1 and 2, whose comments are appended below. In the light of their advice I regret to inform you that we cannot publish your manuscript in Communications Earth & Environment.

As you will see that the reviewers continue to raise substantive concerns, both regarding your methodology and also regarding the novelty of your work compared to the body of work in the literature. Unfortunately, these reservations are sufficiently important to preclude publication of this study in Communications Earth & Environment.

We are committed to providing a fair peer-review process. Please feel free to contact us if you feel there is a misunderstanding or wish to discuss the decision in more detail.

I am sorry that we cannot be more positive on this occasion and thank you for the opportunity to consider your work.

Best regards,

Heike Langenberg, PhD
Chief Editor
Communications Earth & Environment

On Twitter: @CommsEarth

Reviewers' comments:

Reviewer #1 (Remarks to the Author):

I appreciate that the authors have done a lot of work to address my comments, as well as the other reviewers' comments. However, now that more detail about the method used has been provided, I see a major flaw with this approach, and that is the fact that the quantity of interest, ECS, is also what is used to derive the model weights.

Since we don't know what the true ECS is, as it is not an observable quantity, any target ECS distribution is just a good guess. If I understand this approach correctly, the weights essentially nudge the model average ECS value towards the target ECS. This is circuitous. If you picked a high target ECS, the method would downweight models that have low ECS. But because the authors believe that the "hot" models are the problem, they have chosen a ECS value (and distribution) to downweight models with high ECS.

I believe this method would have value if the weights were based on a target quantity that is observable, and independent. In choosing such a target, the authors would need to take into account the large body of existing work on model performance metrics and emergent constraints.

Reviewer #2 (Remarks to the Author):

The authors have addressed most of my comments and those of the other reviewers. I have however two remaining concerns.

1. It is still not clear to me what is going on with the TCR test. The authors use the word "metric" and

I don't know what they mean. There are two metrics: the calibration metric, and a target (predicted) quantity. The weights are assigned so as to make the posterior PDF of the calibration metric as close as possible to a target "truth" distribution; then these weights can be used to determine a pdf for any target quantity. What is not clear to me in the TCR results is whether TCR was used as a calibration, or a target. The language is vague but implies that TCR was used "instead of ECS" as the calibration. However the methods section says the weights are calibrated to ECS--if there will also be calibration to TCR, it should leave this general and then say the calibration metric can be either ECS or TCR. The TCR results presented suggest to me, as I stated in my previous review, that the weights must have been calibrated to ECS, not TCR; otherwise I cannot understand how the posterior PDF would be so far off the target one.

2. The paper now includes lots of new information on previous studies, including several Bayesian model averaging ones. So what is new here? The new section does not say what is new in this approach, only what its advantages are relative to non-Bayesian approaches. If Bayesian approaches have already been used, why do we need this study? I don't think it should be published unless there is some new advance.

Reviewers' comments:

Reviewer #1 (Remarks to the Author):

I appreciate that the authors have done a lot of work to address my comments, as well as the other reviewers' comments. However, now that more detail about the method used has been provided, I see a major flaw with this approach, and that is the fact that the quantity of interest, ECS, is also what is used to derive the model weights.

We are sincerely appreciative of the reviewer for the time spent assessing our manuscript. We attempt to provide some additional details that may help with placing our work within the scope of the broader community, and that may shed some additional light on our main objectives. We hope that after seeing the changes to our manuscript, as well as the additional details laid out in this letter, that the reviewer sees and accepts the value of our presented work.

Since we don't know what the true ECS is, as it is not an observable quantity, any target ECS distribution is just a good guess. If I understand this approach correctly, the weights essentially nudge the model average ECS value towards the target ECS. This is circuitous. If you picked a high target ECS, the method would downweight models that have low ECS. But because the authors believe that the "hot" models are the problem, they have chosen a ECS value (and distribution) to downweight models with high ECS.

I believe this method would have value if the weights were based on a target quantity that is observable, and independent. In choosing such a target, the authors would need to take into account the large body of existing work on model performance metrics and emergent constraints.

We again thank the reviewer for the constructive comments and for the valuable insight. We would like to provide a quote from the Intergovernmental Panel on Climate Change (IPCC) Working Group 1 (WG1) Assessment Report 6 (AR6) Technical Summary (https://www.ipcc.ch/report/ar6/wg1/chapter/technical-summary/):
*“Constraints on equilibrium climate sensitivity (ECS) and transient climate response (TCR) (see Glossary) are based on four main lines of evidence: feedback process understanding, climate change and variability seen within the instrumental record, paleoclimate evidence, and so-called ‘emergent constraints’, whereby a relationship between an observable quantity and either ECS or TCR established within an ensemble of models is combined with observations to derive a constraint on ECS or TCR. In reports up to and including the IPCC Third Assessment Report, ECS and TCR derived directly from Earth System Models (ESMs) were the primary line of evidence. However, since AR4, historical warming and paleoclimates provided useful additional evidence (Figure TS.16a). This Report differs from previous reports in not directly using climate model estimates of ECS and TCR in the assessed ranges of climate sensitivity.”*

Therefore, the reviewer's concern of using ECS as a metric for weighting the climate models may be due to a misunderstanding of the nature of our objectives and methods. We are attempting to derive a distribution of ESMs that most closely aligns with the scientific community's best estimate (which includes uncertainty) of the true, yet unobserved ECS. We have not 'chosen' an ECS value as the reviewer suggests. Rather, based on multiple lines of independent evidence, which importantly do not include the ESMs themselves, we are attempting to derive a distribution of ESM output that most closely matches the scientific community's assessment of ECS. Respectfully, we disagree that this is circuitous logic, and instead we are doing what the community is recommending.

The ECS distribution, which we take directly from the IPCC AR6 WG1 summary, is based on multiple lines of evidence and does not include results from the CMIP6 ESMs. Thus, it is an independent estimate of the ECS quantity, and our approach is a valid one for deriving a weighted ESM ensemble that better reflects our physical understanding of the earth's climate system and its sensitivity to atmospheric greenhouse gas concentrations. To this end, we can provide numerous recent publications that utilize information on the best estimate of ECS to address the physical plausibility issues apparent in aspects of the CMIP6 ensemble (i.e., the 'hot model' problem) that is known in the scientific community to exist in the CMIP6 ensemble (e.g., https://doi.org/10.5194/hess-2023-47).

We are taking this a step further by applying a state-of-the-science approach, the Bayesian Model Averaging method (BMA), that includes detailed information on ECS and its uncertainty distribution when generating a weighted ensemble average. The special benefit of BMA is that the ESM ensemble member 'weights' are derived probabilistically in a Bayesian approach rather than the typical ad hoc approach (such as suggested in model culling techniques in Hausfather et al. 2022). This is a novel strategy applied to this vexing problem of known, and consequential physical limitations in ESMs but which nonetheless are state-of-the-science numerical models. Our BMA approach allows all the models in the ensemble to 'have a voice' and provide information to the model average. Therefore, this strategy is an advancement from the ad hoc approach of simply eliminating 'hot models' from the ensemble, which is what other studies have done and are currently doing.

We have provided some of these points in the new version of the manuscript (e.g., Lines 51-54, 60-62, 93-99). We hope that we have addressed the reviewer's previous concerns and we thank the reviewer once more for the time spent on this paper.

Reviewer #2 (Remarks to the Author):

The authors have addressed most of my comments and those of the other reviewers. I have however two remaining concerns.

We are sincerely appreciative of the reviewer for the time spent assessing our manuscript. We attempt to provide some additional details that may help with placing our work within the scope of the broader community, and that may shed some additional light on our main methods and objectives. We hope that after seeing the changes to our manuscript, as well as the additional details laid out in this letter, that the reviewer sees and accepts the value of our presented work.

1. It is still not clear to me what is going on with the TCR test. The authors use the word "metric" and I don't know what they mean. There are two metrics: the calibration metric, and a target (predicted) quantity. The weights are assigned so as to make the posterior PDF of the calibration metric as close as possible to a target "truth" distribution; then these weights can be used to determine a pdf for any target quantity. What is not clear to me in the TCR results is whether TCR was used as a calibration, or a target. The language is vague but implies that TCR was used "instead of ECS" as the calibration. However the methods section says the weights are calibrated to ECS--if there will also be calibration to TCR, it should leave this general and then say the calibration metric can be either ECS or TCR. The TCR results presented suggest to me, as I stated in my previous review, that the weights must have been calibrated to ECS, not TCR; otherwise I cannot understand how the posterior PDF would be so far off the target one.

We again thank the reviewer for the constructive comments and for the valuable insight. We have clarified the difference between the ECS and the TCR investigations in the methods section. We now lay out additional details in the methods section explaining the separate test for the TCR analysis.

The two ‘metrics’, ECS and TCR, are different yet similar in nature. In our paper, we apply the model weighting on both ECS and TCR separately, because the community recommendation is to focus on both ECS and TCR, if possible. This is why we have results for applying the model weighting on both these climate ‘metrics’. Our main set of results are for applying the BMA method on the ECS metric, which is why we present the results for ECS in the main text. However, since the community also recommends the investigation of TCR, we decided to replicate/mimic the results generated for ECS, but for the TCR metric, and present those results in the supplementary section. Although the results between ECS and TCR are not identical, the main conclusion and story behind the results does not change much. In essence, models with implausible climate sensitivity (whether that is defined through ECS or TCR values that are out of range), tend to get lower BMA weights and therefore provide less information to the overall model average, which in turn results in less exaggerated future climate projections. Lastly, by applying BMA on either ECS or TCR, all models in the ensemble are still considered, rather than rejected, which is an advancement from current methods (e.g., Hausfather et al., 2022).

2. The paper now includes lots of new information on previous studies, including several Bayesian model averaging ones. So what is new here? The new section does not say what is new in this approach, only what its advantages are relative to non-Bayesian approaches. If Bayesian approaches have already been used, why do we need this study? I don't think it should be published unless there is some new advance.

We again thank the reviewer for this constructive comment. We would like to highlight that a major aspect of novelty in our work is the use of BMA for a policy-relevant case study, which to the best of our knowledge has not been done yet. The novel strategy of using BMA in a policy-relevant case study to allow all the models in the ensemble to 'have a voice' and provide information to the estimated model average of ECS has not been done before. Therefore, this strategy is an advancement from simply eliminating 'hot models' from the ensemble, which is what other studies have done and are currently doing (e.g., https://doi.org/10.5194/hess-2023-47). Although BMA is not new, it has not been applied in a highly policy relevant context as shown in our work. Furthermore, BMA gives us the best chance to retain information from all climate models. So, it is in direct response to the rather heavy-handed approach of model-culling (e.g., Hausfather et al., 2022), which is also not a new approach, and yet was deemed worthy to be published in Nature, precisely because of its policy relevance. We now explain this in the text on Lines 93-99.

Given the recent focus on the high sensitivity of some CMIP6 models, a plausible outcome in the community is that whichever approach is adopted to address the 'hot model' issue is then applied to projections of other fields (e.g., precipitation). Thus, our procedure is partially motivated by a desire to preserve as much information as possible from the ensemble that is consistent with our physical understanding of the earth's climate system, rather than out-right rejecting certain models, and therefore potentially rejecting useful information from the projections.

We have made additional comments in the manuscript (e.g. Lines 93-99, Lines 300-303) to help address the reviewer's concerns. We have also made changes to our manuscript that can hopefully shed additional light on our objective and methods. We hope the new additions and clarifications further show the value of this work and how it fits within the broader community effort to derive accurate and reliable projections of climate change from multi-model ensembles. We hope that we have addressed the reviewer's previous concerns and we thank the reviewer once more for the time spent on this paper.

Dear Dr Massoud,

Your revised manuscript titled "Bayesian weighting of climate models based on climate sensitivity" has now been seen by our original two reviewers. Reviewer 1 had no further comments for the authors, and reviewer 2's comments are appended below. In the light of their advice I regret to inform you that we cannot publish your manuscript in Communications Earth & Environment.

You will see that the reviewer 2 still raises apparently serious concerns with your proposed method for weighting climate models, and suggests that it does not work. Unfortunately, these reservations are sufficiently important to preclude publication of this study in Communications Earth & Environment.

I am sorry that we cannot be more positive on this occasion and thank you for the opportunity to consider your work.

Best regards,

Heike Langenberg, PhD
Chief Editor
Communications Earth & Environment

On Twitter: @CommsEarth

Reviewers' comments:

Reviewer #2 (Remarks to the Author):

I think most of the concerns from the reviewers have been addressed, but I remain very concerned about the authors' results and suggest this paper not be published until they can address my final point below. In short, I don't think their calculation is working correctly.

First, with respect to the other reviewer's concern, I agree with the authors that this reviewer misunderstood the goals of the study.

With respect to my concern about novelty, I now see that the novelty of this study is to apply Bayesian model weighting to solve the "hot model" problem (or more generally to calibrate a set of models to a desired distribution of sensitivity), whereas it has previously been used to calibrate directly to observations (e.g. historical temperature) while the "hot model" problem has been addressed only by simple truncation methods. The authors however do not say this--why not? Instead they now say their approach as more "policy relevant" which I think is meaningless and inappropriate -- please explain methodologically what is new. I would say that the value here is that ECS and TCR are much more relevant for future climate change than are past historical temperature observations, so constraining models based on sensitivity will be a lot better (provided the ECS/TCR distributions are strongly informed by relevant observations, which the revised manuscript now explains).

With respect to my confusion about TCR vs. ECS, in the response to reviews the authors clearly explain that their main analysis calibrates to ECS, but then in the Supplement they present one calibrated to TCR as an alternative. OK that makes sense. But the revised manuscript is still unclear about this. The new statement at lines 240-242 makes it sound like they are calibrating to both ECS and TCR at once! The authors need to think about how they word things to avoid ambiguity, and try to be clearer. Why not just tell readers what you told us?

Finally and most importantly, I remain puzzled and concerned about the TCR result in the Supplement. The authors have now confirmed that this result is for calibration to a target TCR distrivbution, but the

BMA result (Fig. S1) is not closer to the target than the original raw model distribution; true, the mean is corrected, but the variance is now much too narrow whereas the original GCM spread was about right. This means the method is not working! It will underestimate any future uncertainty that depends on TCR. I can't believe BMA would be this bad and suspect the authors have done something wrong (the better performance on ECS could therefore be accidental).

If it can go this wrong, it should not be used. A simple kernel-density estimation approach (where each GCM is weighted by the ratio of the target density to the local sample density of models in TCR space) would perform far better than this.

Another problem is that in Fig. S2 the caption states that "hotter" models were downweighted. But Fig. S1 shows that the GCM TCR distribution (unlike the ECS one) is actually too low, so they should be downweighting the low-TCR models not the high ones. It also says this is linear but in Panels B and D it is a curved function. The method seems to be attaching most weight to the central members, but this is not what we want.

Reviewers' comments:

Reviewer #2 (Remarks to the Author):

I think most of the concerns from the reviewers have been addressed, but I remain very concerned about the authors' results and suggest this paper not be published until they can address my final point below. In short, I don't think their calculation is working correctly.
We again thank the reviewer very much for taking the time to review our paper. We want to clarify the results for the TCR metric, and hope that the corrections and edits we now include help provide some insight on that part of the analysis.

First, with respect to the other reviewer's concern, I agree with the authors that this reviewer misunderstood the goals of the study.
Thank you, we agree and hope that after the last revisions the other reviewer now sees the value of this work.

With respect to my concern about novelty, I now see that the novelty of this study is to apply Bayesian model weighting to solve the "hot model" problem (or more generally to calibrate a set of models to a desired distribution of sensitivity), whereas it has previously been used to calibrate directly to observations (e.g. historical temperature) while the "hot model" problem has been addressed only by simple truncation methods. The authors however do not say this--why not? Instead they now say their approach as more "policy relevant" which I think is meaningless and inappropriate -- please explain methodologically what is new. I would say that the value here is that ECS and TCR are much more relevant for future climate change than are past historical temperature observations, so constraining models based on sensitivity will be a lot better (provided the ECS/TCR distributions are strongly informed by relevant observations, which the revised manuscript now explains).
Thank you for this comment. We would like to bring attention to the comments in our introduction that are very similar to what the reviewer is mentioning here. On Line 93 in the paper, we write:
*"Applying BMA in this context is a novel strategy which allows all the models in the ensemble to 'have a voice' and provide information to the estimated model average of ECS or TCR. Therefore, this strategy is an advancement from simply eliminating 'hot models' from the ensemble, as other studies have done and are currently doing (see Hausfather et al., 2022; Asenjan et al., 2023)."*

It was after this comment in the paper that we mentioned the policy-relevance. However, we agree with the reviewer that the policy-relevance is less important for this paper and have therefore removed those statements. We now instead focus even more on the comments made above, and now state on Line 97 of the new manuscript:
*"Furthermore, the novelty of this study is to apply Bayesian model weighting to solve the 'hot model' problem, or more generally to calibrate a set of models to a desired distribution of sensitivity, whereas previous works that have applied model weighting have calibrated directly to observations such as historical temperature or precipitation (e.g., Sanderson et al., 2017;*

*Massoud et al., 2020; Wootten et al., 2023). The value here is that ECS and TCR are much more relevant for future climate change than are past historical temperature observations, so constraining models based on sensitivity will be more meaningful in capturing simulated future climates (provided that the ECS and TCR distributions are strongly informed by relevant observations and evidence)."*

With respect to my confusion about TCR vs. ECS, in the response to reviews the authors clearly explain that their main analysis calibrates to ECS, but then in the Supplement they present one calibrated to TCR as an alternative. OK that makes sense. But the revised manuscript is still unclear about this. The new statement at lines 240-242 makes it sound like they are calibrating to both ECS and TCR at once! The authors need to think about how they word things to avoid ambiguity, and try to be clearer. Why not just tell readers what you told us?
We thank the reviewer for this comment and apologize for any confusion that may have been caused by the text. We tried to clarify that the tests were separate, the main test being for ECS, and then an additional test done separately for TCR. To avoid any confusion, we now re-word these statements on Line 239 in the main text to read as follows:
*"Our approach makes use of the ECS metric as a main target for fitting the BMA model average. However, other metrics can be useful in this regard as well, such as the Transient Climate Response (TCR), which is the mean global warming predicted to occur around the time of doubling CO2 in Earth System Models (ESM) runs for which atmospheric CO2 concentration is prescribed to increase at 1% per year. Based on multiple lines of evidence (Scafetta 2021), TCR has an assessed likely range of 1.4°C to 2.2°C (c.f. the IPCC AR6 WG1 technical summary). Therefore, we apply the BMA on the assessed TCR distribution to produce a set of model weights that optimize the model-based TCR estimate.*

*We produced a second set of results to mirror the outcome from applying BMA on the ECS metric (the results of which are shown in the main text, Figures 1-3, Table 1), but we did so for the TCR metric (the results of which are shown in the supplement, Figures S1-S3, Table 1). Similar to Figure 1 that is based on the ECS metric, supplemental Figure S1 shows results based on the TCR metric, including individual CMIP6 model TCR scores, the assessed TCR distribution, and the estimated BMA model weights and corresponding TCR distributions. Supplemental Figure S2 shows the dependence scores based on applying BMA on the TCR metric, as well as how these scores relate with the BMA model weights and TCR scores. The final figure in the supplement, Figure S3, shows the projected increase in global mean surface temperature (°C) for the different SSP scenarios considered and the different model averaging methods used, and panel B of this figure includes both the ECS and TCR based projections for comparison.*

*Like the results of applying BMA on ECS, doing so on the TCR metric produces a weighted mean projection that is more in line with all lines of evidence compared to that produced using the raw CMIP6 multi-model mean…"*

Finally and most importantly, I remain puzzled and concerned about the TCR result in the Supplement. The authors have now confirmed that this result is for calibration to a target TCR distrivbution, but the BMA result (Fig. S1) is not closer to the target than the original raw model

distribution; true, the mean is corrected, but the variance is now much too narrow whereas the original GCM spread was about right. This means the method is not working! It will underestimate any future uncertainty that depends on TCR. I can't believe BMA would be this bad and suspect the authors have done something wrong (the better performance on ECS could therefore be accidental).

If it can go this wrong, it should not be used. A simple kernel-density estimation approach (where each GCM is weighted by the ratio of the target density to the local sample density of models in TCR space) would perform far better than this.
We again thank the reviewer for this comment. We would like to point out there was an error in the figure caption from the last submission. In the caption, we mixed up the labeling of the TCR target distribution and the BMA posterior distribution of TCR. We have now corrected this error. Furthermore, we have updated the figure to show only curves for all the probability distributions (instead of histograms for some and curves for others). This allows for a better visual comparison of the different pdf curves. Lastly, and most importantly, the reviewer is correct in that the previous results showed that the BMA posterior for TCR was indeed too narrow and did not fully represent the uncertainty of the assessed TCR distribution. To take care of this issue, we relaxed the constraints in the BMA setup (i.e. we allowed the likelihood function to accept more samples). This small edit in the BMA setup now allows for a much better fit to the distribution (please see updated Figure S1). This is the benefit of using a framework like BMA, which allows flexibility in estimating the model weights.

The new set of BMA weights for the TCR metric are not much different than the previous weights since this edit to the BMA set up was very minor. The corresponding future projections are therefore also not much different. The only major change as a result of this new set up is that the assessed TCR distribution and the BMA posterior TCR distribution are more closely matched (Figure S1C). In essence, the same conclusion can be reached, which is BMA offers a more robust alternative to weighting models compared to simple model truncation or even the raw multi-model mean. This can especially be seen in Figure S3B, where we show and compare the climate results from both the ECS and TCR-based projections.

Furthermore, we do agree with the reviewer that other approaches (such as KDE) can be utilized in studies like this. We now provide a concluding paragraph in the manuscript that addresses KDE and other model averaging approaches and points out the difference between BMA and these approaches. On Line 352 we now state:
"*In conclusion, as an alternative to simple model culling, we recommend the use of any model averaging approach that allows the user to justify non-zero weights on all members of the model ensemble. In this study, we have focused on ECS and TCR to address the hot model problem. Previous studies targeted towards specialized impact studies focused on other phenomena such as drought (Gonzalez Cruz et al., 2020) or used an expert but arbitrary selection of observed mean quantities (Sanderson et al., 2017) targeted towards a skillful general-purpose ensemble. However, the unreasonably large range of model climate sensitivities in the CMIP6 ensemble requires attention to model trends. Here we have used a BMA approach, but previous statistically based model weighting studies have used a simple*

*kernel-density estimation (KDE) approach (Gonzalez Cruz et al., 2020) or the Sanderson approach (Sanderson et al., 2017). With KDE, each ESM is weighted by the ratio of the target density to the local sample density of models in ECS or TCR space. The Sanderson approach implements the predetermined skill and independence scores of each model when estimating the model weights.*

*We specifically recommend BMA to apply model averaging when feasible, since the total-order distribution of the model weights (and therefore the total-order distribution of the projected climate change signal) is estimated with BMA, whereas with the KDE or Sanderson approaches, only the first-order distribution of the model weights is estimated. In other words, the BMA method allows for the estimation of the entire distribution of model weights and corresponding climate change signals (and therefore the full uncertainty distribution), where the KDE or Sanderson approaches simply nudge the model weights in the direction of the optimal set of weights. Furthermore, regarding model independence, the BMA method allows for the estimation of model independence during post-processing and provides information on dependent model weights that are used for future projections. This is important because we want to know the dependence of each models' contribution to the estimated model average (and therefore the dependence of each models' contribution to the projected climate change signal with BMA). In comparison, the Sanderson approach uses predetermined information to estimate model independence and is based on the model output space, not the model weights space. This is different than the BMA estimation of model dependence because the Sanderson approach uses information on which models have similar model genealogy, shared code and parameterizations, or similar model outputs, while BMA provides information on which models have posterior weights that are correlated together (and therefore which models are dependently contributing to the projected climate change signal). What is more, the KDE approach does not estimate model independence. For these reasons, we recommend the use of the BMA method for model averaging studies in climate sciences."*

Another problem is that in Fig. S2 the caption states that "hotter" models were downweighted. But Fig. S1 shows that the GCM TCR distribution (unlike the ECS one) is actually too low, so they should be downweighting the low-TCR models not the high ones. It also says this is linear but in Panels B and D it is a curved function. The method seems to be attaching most weight to the central members, but this is not what we want.
We thank the reviewer for this final comment. We would like to explain that some models are indeed 'too hot', but there are also models in the ensemble that are 'not hot enough', meaning they have an ECS or TCR value that is lower than the assessed range. So the BMA method downweighs models that are either 'too hot' or 'not hot enough'. This is displayed in the scatter plots in Figure 2B-D and Figure S2B-D, which show the highest weights are applied for those models with ECS and TCR values near the peak (near 3°C for ECS and 1.8°C for TCR), and the weights do drop almost linearly for ECS and TCR values that are higher or lower than the peak value. However, even though the weights drop for some models, there are no models that have weights that are too low or too high. In other words, all models generally have weights that allow them to 'have a voice', with weights in the range of ~0.03-0.08. For comparison with the equal weights approach (i.e., a simple model mean), all models would have a weight of 1/16 or

0.0625 if equal weights were applied.  Again, this is the benefit of using BMA over the other mentioned methods since all models can still provide significant information to the model average when using the BMA method. This is now explained in the text on Line 263.

Furthermore, Table 1 now shows which models have either ‘higher’ or ‘lower’ ECS and TCR values compared to their assessed ranges. And according to Table 1, we see that there are more models that have a high ECS or TCR value than models that have low values. So, the ’hot model’ problem does indeed exist in the CMIP6 ensemble, and this can be seen when looking at either the ECS or the TCR metrics. Overall, our framework shows how to deal with this issue without having to reject any models, but instead by combining the information from all available models.

Dear Dr Massoud,

Your revised manuscript titled "Bayesian weighting of climate models based on climate sensitivity" has now been seen by our reviewer, whose comments appear below. In light of their advice we are delighted to say that we are happy, in principle, to publish a suitably revised version in Communications Earth & Environment, provided you can address the remaining comments from the reviewer in full. If appropriate, the manuscript will be published under the open access CC BY license (Creative Commons Attribution v4.0 International License).

We therefore invite you to revise your paper one last time to address the remaining concerns of our reviewers. At the same time we ask that you edit your manuscript to comply with our format requirements and to maximise the accessibility and therefore the impact of your work.

EDITORIAL REQUESTS:

Please review our specific editorial comments and requests regarding your manuscript in the attached "Editorial Requests Table".

*****Please take care to match our formatting and policy requirements. We will check revised manuscript and return manuscripts that do not comply. Such requests will lead to delays. *****

Please outline your response to each request in the right hand column. Please upload the completed table with your manuscript files as a Related Manuscript file.

If you have any questions or concerns about any of our requests, please do not hesitate to contact me.

SUBMISSION INFORMATION:

In order to accept your paper, we require the files listed at the end of the Editorial Requests Table; the list of required files is also available at https://www.nature.com/documents/commsj-file-checklist.pdf .

OPEN ACCESS:

Communications Earth & Environment is a fully open access journal. Articles are made freely accessible on publication under a <a href="http://creativecommons.org/licenses/by/4.0" target="_blank"> CC BY license</a> (Creative Commons Attribution 4.0 International License). This license allows maximum dissemination and re-use of open access materials and is preferred by many research funding bodies.

For further information about article processing charges, open access funding, and advice and support from Nature Research, please visit <a href="https://www.nature.com/commsenv/article-processing-charges">https://www.nature.com/commsenv/article-processing-charges</a>

At acceptance, you will be provided with instructions for completing this CC BY license on behalf of all authors. This grants us the necessary permissions to publish your paper. Additionally, you will be asked to declare that all required third party permissions have been obtained, and to provide billing information in order to pay the article-processing charge (APC).

Please use the following link to submit the above items:
[Link redacted]

** This url links to your confidential home page and associated information about manuscripts you may have submitted or be reviewing for us. If you wish to forward this email to co-authors, please

delete the link to your homepage first **

We hope to hear from you within two weeks; please let us know if you need more time.

Best regards,

Heike Langenberg, PhD
Chief Editor
Communications Earth & Environment

On Twitter: @CommsEarth

REVIEWERS' COMMENTS:

Reviewer #2 (Remarks to the Author):

The authors have addressed the issues I raised, but the response has raised other issues, plus I now see another problem, #2 below (maybe it was the same before and I missed it, if so, sorry about that).

1. The authors claim their method is "robust," but this is contradicted by the fact that their posterior PDF changed quite a lot due to fiddling with some parameter for which they do not seem to have a justification. This requires more discussion -- for example, does it mean that various values of this parameter must be tried and the posterior PDF checked against the target in order to arrive at a good result? This is the sort of tuning approach often needed in machine learning as well. It needs to be explained, if anyone is going to use this approach!

2. The target PDF shown in black in Fig. 1 -- where did this come from? The manuscript doesn't say. Why wasn't a PDF from Sherwood et al. (2020) or AR6 used as the target? The PDF shown is much narrower and is not consistent with the likely range quoted in those sources and noted in the manuscript text, in fact, it shows a nearly 100% chance of being in the 2.5-4C range whereas the current view is that there is only a 66% chance of being in this range. This doesn't really matter if the only goal is to demonstrate the method, but the paper is also making statements about future temperature rise based on the results, which will be affected by the PDF.

3. Their results still don't look consistent, and this is particularly evident for the TCR test (but also I think for ECS). I cannot believe that the nearly uniform weighting shown in Fig. S1B could possibly produce the large reduction in tail probabilities shown in Fig. S1C. For example the latter shows about a five-fold reduction in the probability density for TCR=2.0, but the weights attached to the Canada and IPSL models with the highest TCR values are at most maybe 20% lower than average. How can reducing the weight of high-TCR models by only 20% reduce the probability of high values fivefold? I am also suspicious of the large sensitivity of the result the authors now report (point 1) from only adding a few more sample members. It should not be that sensitive. Perhaps the authors should do some tests starting with only two models, and adding one at a time, to carefully check whether the results make sense. So far I am not convinced that this is a good approach....
